# Neurogenesis of medium spiny neurons in the nucleus accumbens continues into adulthood and is enhanced by pathological pain

**DOI:** 10.1038/s41380-020-0823-4

**Published:** 2020-07-01

**Authors:** Diego García-González, Ionut Dumitru, Annalisa Zuccotti, Ting-Yun Yen, Vicente Herranz-Pérez, Linette Liqi Tan, Angela Neitz, José Manuel García-Verdugo, Rohini Kuner, Julieta Alfonso, Hannah Monyer

**Affiliations:** 1 Department of Clinical Neurobiology, University Hospital Heidelberg and German Cancer Research Center, Heidelberg, Germany; 2 Taiwan International Graduate Program in Molecular Medicine, National Yang-Ming University and Academia Sinica, Taipei, Taiwan; 3 Laboratory of Comparative Neurobiology, Cavanilles Institute, University of Valencia, CIBERNED, Valencia, Spain; 4 Predepartamental Unit of Medicine, Faculty of Health Sciences, Universitat Jaume I, Castelló de la Plana, Spain; 5 Institute of Pharmacology, Heidelberg University, Heidelberg, Germany; 6 Present Address: Biotech Research and Innovation Centre (BRIC), Faculty of Health, University of Copenhagen, Copenhagen N, Denmark; 7 Present Address: Department of Cell and Molecular Biology, Karolinska Institute, Stockholm, Sweden

**Keywords:** Neuroscience, Stem cells, Depression

## Abstract

In mammals, most adult neural stem cells (NSCs) are located in the ventricular–subventricular zone (V-SVZ) along the wall of the lateral ventricles and they are the source of olfactory bulb interneurons. Adult NSCs exhibit an apico-basal polarity; they harbor a short apical process and a long basal process, reminiscent of radial glia morphology. In the adult mouse brain, we detected extremely long radial glia-like fibers that originate from the anterior–ventral V-SVZ and that are directed to the ventral striatum. Interestingly, a fraction of adult V-SVZ-derived neuroblasts dispersed in close association with the radial glia-like fibers in the nucleus accumbens (NAc). Using several in vivo mouse models, we show that newborn neurons integrate into preexisting circuits in the NAc where they mature as medium spiny neurons (MSNs), i.e., a type of projection neurons formerly believed to be generated only during embryonic development. Moreover, we found that the number of newborn neurons in the NAc is dynamically regulated by persistent pain, suggesting that adult neurogenesis of MSNs is an experience-modulated process.

## Introduction

Most forebrain neurons are generated from radial glia (RG) stem cells located in the ventricular zone (VZ) of the cerebral ventricles in the embryonic brain [1–3]. As embryonic RG extend a long basal process from their cell body in the VZ toward the pial surface, they also serve as a guide and scaffold for migrating neuroblasts that disperse from the VZ to reach their specific locations in the brain [4–6]. As development proceeds, the embryonic cerebral ventricles transform into the adult lateral ventricles that retain a fraction of neural stem cells (NSCs) in the ventricular–subventricular zone (V-SVZ) [7, 8]. Adult NSCs originate from embryonic RG cells [9], and function as neural progenitors throughout life [10]. These adult neural progenitors exhibit RG traits, including an apical primary cilium exposed to the lateral ventricle and an elongated basal process that contacts blood vessels [11, 12].

In rodents, cells born in the SVZ of the adult lateral ventricles migrate in chains along the rostral migratory stream (RMS) to reach the olfactory bulb (OB) where, upon maturation, they develop a GABAergic phenotype [13, 14]. In addition, a fraction of postnatally born neuroblasts exits the V-SVZ or RMS and gives rise to distinct subpopulations of small-sized GABAergic interneurons that integrate into regions other than the OB, including the prefrontal cortex and striatal structures such as the olfactory tubercle and the caudate putamen nuclei [15–18].

Despite the close anatomical association between the anterior–ventral (av)V-SVZ and the nucleus accumbens (NAc), a major component of the ventral striatum, adult neurogenesis in the NAc has received little attention ever since it was first proposed almost 50 years ago [15]. The presence of neuroblasts in the NAc together with glial fibers originating in the avV-SVZ were first reported in the mouse brain [19]. It is not known whether the glial fibers in this brain region influence neuroblast migration to the NAc nor whether the neuroblasts integrate into the local NAc circuitry. The NAc is populated by two main neuronal types, namely GABAergic projection neurons, known as medium spiny neurons (MSNs) (∼95%), and local circuit interneurons [20]. Previous studies demonstrated that MSNs are generated in the ventral lateral ganglionic eminence (LGE) during embryonic development and neurogenesis of MSNs has been considered complete before birth [21–23].

The V-SVZ contains distinct spatial microdomains that generate specific subtypes of interneurons after birth [8, 24–27]. Prompted by the fact that the dorsal NAc envelops the avV-SVZ, a highly active neurogenic microdomain of the V-SVZ, we investigated adult neurogenesis in the mouse ventral striatum. We used a set of in vivo approaches to demonstrate that young neurons generated in the adult avV-SVZ exit their site of origin. They are guided by long RG-like processes extending from the SVZ toward the NAc and differentiate into MSNs that integrate into preexisting circuits. Furthermore, our results showed that the extent of NAc neurogenesis is environmentally regulated, as exposure to chronic pain conditions affected the number of newborn MSNs in this brain area.

## Results

### Neuroblast migrate radially from the SVZ to the NAc in adult mice

**Fig. 1:**
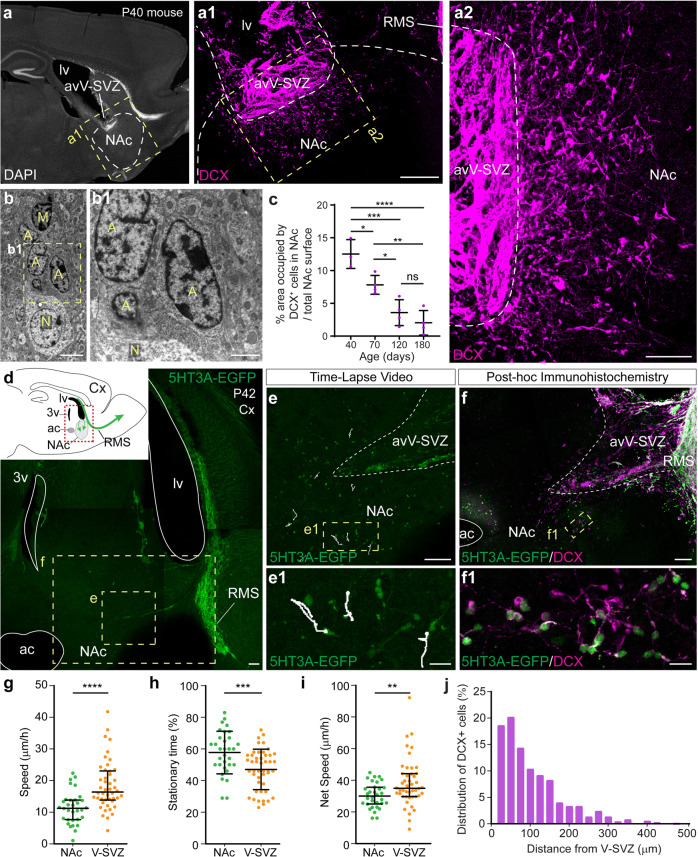
Newborn neuroblasts migrate from the V-SVZ towards the NAc in the adult mouse brain and their numbers decrease with age. **a** Sagittal brain section from a P40 wild-type mouse, immunostained for DAPI. **a1**, **a2** Magnifications of the inset marked in squared yellow dashed outlines in **a** and **a1**, respectively, and show a density gradient of DCX+ neuroblasts from the anterior–ventral V-SVZ to the dorsal NAc. **b** TEM images of neuroblasts (A) in close vicinity to a mature neuron (N) and a microglia cell (M) in the NAc. **c** The surface occupied by DCX+ neuroblasts in the NAc over the total NAc surface decreases with age from P40 to P180 (one-way ANOVA with Bonferroni’s multiple comparison post hoc test; mean ± SD; ****p < 0.0001; ***p < 0.001; **p < 0.01; *p < 0.05). **d** Live-imaging image reconstruction of a sagittal brain section from a P42 5HT3A-EGFP mouse. Inset: schematic of the anatomical region, green arrows illustrate the migratory routes followed by neuroblasts, the dashed red rectangle denotes the area in the picture. **e** Image of the V-SVZ-NAc (area indicated with the small yellow dashed square in **d**) after 240 min of recordings. Supplementary Movie 1 was recorded from this region. White lines illustrate the trajectory of recorded cells and dots indicate the end of the trajectory. **e1** Detailed image from the area indicated with the yellow dashed rectangle in **e**, containing 5HT3A-expressing neuroblasts whose trajectories were tracked. **f** Post hoc immunochemistry performed on the same brain section used in d-e to ascertain DCX expression of 5HT3A+ migrating neuroblasts. The corresponding brain area is indicated with the big yellow dashed rectangle in **d**. **f1** High-magnification image from the area indicated with the yellow dashed box in **f**, showing that 5HT3A+ migrating neuroblasts also express DCX. **g** Speed of 5HT3A-expressing neuroblasts migrating in the NAc (green dots) or within the V-SVZ (orange dots) (***p < 0.0001; Mann–Whitney test; median ± IQR), measured in acute brain slices. **h** Percentage of time that 5HT3A+ neuroblasts spent in stationary (non-moving) state when migrating in the NAc or in the V-SVZ (***p = 0.0006; Mann–Whitney test; median ± IQR). **i** Net speed of 5HT3A+ neuroblasts migrating in the NAc or V-SVZ, calculated as migration velocity excluding time spent in stationary state (**p = 0.0077; Mann–Whitney test; median ± IQR). **g–i** *p*-values are indicated in the figure; the total number of tracked cells was 32 in the NAc and 46 in the anterior–ventral V-SVZ (8 slices from 6 mice were used). **j** Relative distribution of DCX+ neuroblasts with respect to the distance from the V-SVZ in P65 wild-type mice (*n* = 756 cells from 4 mice). Abbreviations: 3v, third ventricle; ac, anterior commissure; Cx, cortex; lv, lateral ventricle; NAc, nucleus accumbens; RMS, rostral migratory stream; V-SVZ, subventricular zone. Scale bars in µm: 200 (**a1**), 100 (**a2**, **d**–**f**), 20 (**e1**, **f1**), 5 (**b**), 2 (**b1**). See also Supplementary Figs. 1 and 2.

Immunohistochemical analysis of brain sections at the lateral ventricle level in young mice (P40) revealed that numerous doublecortin-positive (DCX+) young neurons appeared to migrate ventrally from the avV-SVZ towards the NAc (Fig. 1a; for exact delineation of the avV-SVZ, see Supplementary Fig. 1a). We ascertained the neuronal identity of DCX+ cells in the NAc by labeling with PSA-NCAM antibody and ruled out the co-expression of oligodendroglia (Olig2+) and microglia (Iba1+) markers (Supplementary Fig. 1b, c). The neuroblast identity was also confirmed at the ultrastructural level using transmission electron microscopy (TEM) (Fig. 1b). There was a continuous decrease in the area occupied by DCX+ cells in the NAc from P40 to P180 (Fig. 1c and Supplementary Fig. 2a), in agreement with the fact that the rate of postnatal neurogenesis declines with age.

To obtain direct proof that neuroblasts were indeed actively migrating, we performed time-lapse imaging recordings in acute sagittal brain slices. We used 5HT3A-EGFP transgenic mice where SVZ-derived neuroblasts express the fluorescent protein EGFP (enhanced green fluorescent protein) [18, 28] as a tool to visualize young neuroblasts in 6–7-week-old mice. We discerned two populations of 5HT3A-EGFP+ cells in the NAc. One corresponded to mature 5HT3A-EGFP+ interneurons with large somata and complex dendritic branches, and the other to unipolar or bipolar 5HT3A-EGFP+/DCX+ migrating neuroblasts with small-sized cell bodies. 5HT3A-EGFP+ neuroblasts were recorded while migrating within the parenchyma of the ventral striatal area, confirming that there is active migration of young neurons in the NAc of ~P40 mice (Fig. 1d, e and Supplementary Movie 1). Post hoc immunohistochemistry on the same brain sections confirmed that 92.1 ± 3.2 (mean ± SD, *n* = 3 mice) of 5HT3A-EGFP+ migrating cells were indeed DCX-expressing neuroblasts (Fig. 1f and Supplementary Fig. 2b). Once they exited the avV-SVZ, most 5HT3A-EGFP+ cells migrated individually through the NAc. Individual cell-tracking analysis revealed that neuroblasts migrating in the NAc were slower compared with neuroblasts migrating in chains along the V-SVZ (Fig. 1g-i), similar to radial migration in the OB [28, 29]. The idea that neuroblasts detected in the NAc derive from the V-SVZ is further supported by the finding that the number of neuroblasts decreased with increased distance from the V-SVZ (Fig. 1j).

**Fig. 2:**
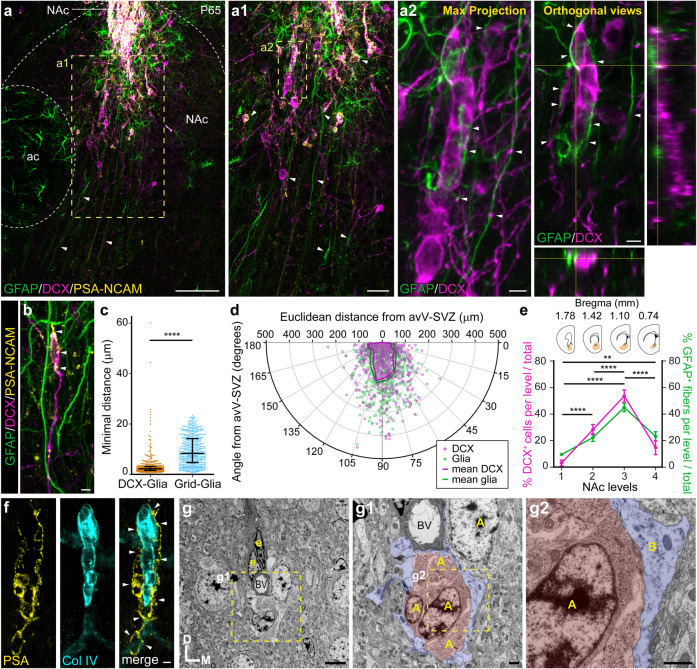
Newborn neuroblasts migrate in a radial-like fashion in the adult NAc. **a** Coronal brain section of a P65 wild-type mouse. DCX+/PSA-NCAM+ neuroblasts exhibit a density gradient from the ventral V-SVZ to the NAc. **a1** High-resolution image from the area indicated with the yellow dashed rectangle in a. **a2** High-magnification (×63) image from the area indicated with the yellow dashed rectangle in **a1**. Left, maximum-projection image showing GFAP+ fibers paralleling DCX+ neuroblasts. Right: single-plane detailed image and orthogonal views showing direct contacts between glial fibers and neuroblasts. **b** High-magnification image showing DCX+/PSA-NCAM+ neuroblasts aligned with GFAP+ glial fibers in P65 wild-type mice. **c** The Euclidean distance between DCX+ neuroblasts and the closest GFAP+ glial fiber in the NAc is shorter than that of a randomly established grid and glial fibers (***p < 0.0001; Mann–Whitney test; median ± IQR). **d** The direction of migrating neuroblasts strongly correlates with the direction of glial fibers in the NAc of 8-week-old wild-type mice. Each dot indicates single DCX+ neuroblasts (magenta) or GFAP+ glial fibers (green). Continuous lines represent the average distribution for both groups considering the Euclidean distance and the angle from the ventral corner of the V-SVZ. **e** Relative distribution of DCX+ neuroblasts (magenta) and GFAP+ (green) fibers at four rostro-caudal levels of the NAc (**p < 0.01; ****p < 0.0001; two-way ANOVA with Bonferroni’s multiple comparison post hoc test; mean ± SD). **f** PSA-NCAM+ neuroblasts migrate following Collagen IV+ blood vessels in the NAc. **g** Pseudo-colored TEM images showing chains of neuroblasts (A) surrounded by an astrocyte (B) that also establishes contact with a blood vessel (BV). Abbreviations: ac, anterior commissure; lv, lateral ventricle; NAc, nucleus accumbens. Scale bars in µm: 100 (**a**), 25 (**a1**), 5 (**a2**, **b**, **f**, **g**), 1 (**g1**, **g2**). See Supplementary Figs. 3–6 and Supplementary Movies 2 and 4.

We next investigated the cellular mechanism by which young neuroblasts disperse from the avV-SVZ into the NAc. We detected long basal glial processes (Vimentin+/GFAP+) originating in the V-SVZ that extended hundreds of micrometers (up to 1 mm) into the ventral striatum (Supplementary Fig. 3a, b). We did not observe, however, long glial fibers in the dorsal striatum or in the septum at all ages that we examined—as evidenced by the DARPP-32/Vimentin co-staining—suggesting a NAc-specific preference (Supplementary Fig. 3a, b). Glial fibers extended a basal end foot process on blood vessels in the NAc, as revealed by labeling with Collagen IV antibody (Supplementary Fig. 4a-e). A small fraction of Vimentin+ glial cells expressed the proliferation marker Ki67, indicative of active cell division (Supplementary Fig. 3c-e). There was a reduction in the number of glial fibrillary acidic protein (GFAP)+/Vimentin+ glial fibers from P40 to P180, consistent with a decrease of neurogenesis that is linked to ageing (Supplementary Fig. 5a-d). Given that the morphology, orientation, and glial nature of these long processes resembled those of embryonic RG fibers, we referred to them as RG-like fibers. High-magnification confocal microscopy revealed that neuroblasts were in close proximity to glial fibers, prompting the hypothesis that the latter might be instructive for neuroblast migration (Fig. 2a, b, Supplementary Fig. 6a, and Supplementary Movies 2–5). To evaluate a possible association between DCX+/PSA-NCAM+ neuroblasts and RG-like fibers, we measured the distance between neuroblasts exiting the avV-SVZ and the nearest GFAP+ glial process in 9-week-old mice. We found that the minimal distance between neuroblasts and glial processes was significantly shorter than if the distribution of neuroblasts was random (Fig. 2a, c). Moreover, we compared the relative position of all DCX+ neuroblasts and GFAP+ glial fibers in a given coronal section (50 µm in thickness) and found a similar distribution of both cell populations, suggesting that RG-like fibers instruct the migration of DCX+ neuroblasts (Fig. 2d). This notion is further supported by the finding that the non-uniform distribution of GFAP fibers along the rostro-caudal axis is paralleled by a similar distribution of neuroblasts (Fig. 2e). Needless to say that additional support for migrating neuroblasts cannot be ruled out given the close proximity both to blood vessels (Fig. 2f, g and Supplementary Movies 3), as well as to myelinated axonal tracts in the striosomes (Supplementary Fig. 6b). Together, these results indicate that young V-SVZ-derived neuroblasts migrate in the adult NAc supported by glial processes and blood vessels.

### Postnatally generated neurons in the NAc develop into MSNs

**Fig. 3:**
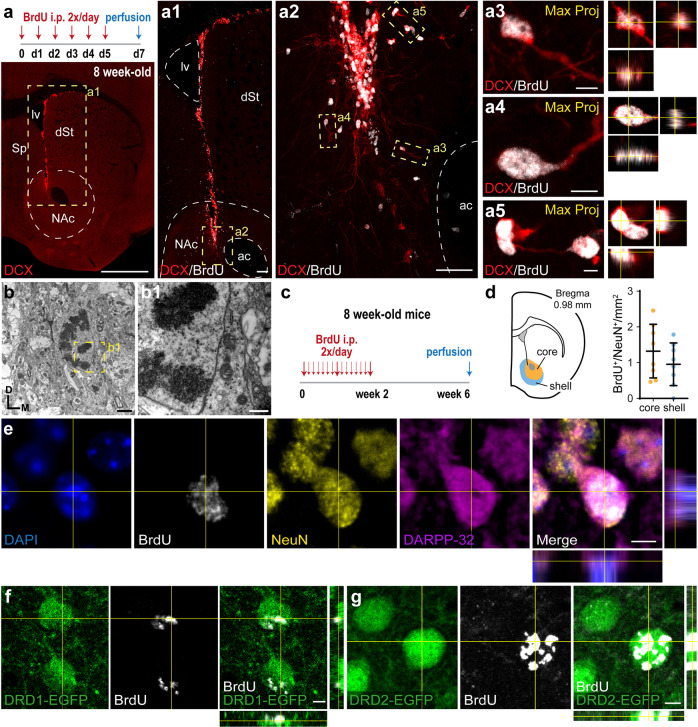
Newborn neurons in the adult NAc. **a** Top: schematic of the experimental protocol. Bottom: representative picture of a coronal brain section from an 8-week-old mouse stained with antibodies against DCX (red), showing neuroblasts in the V-SVZ. Higher magnifications of the boxed areas (dashed yellow lines) are shown on the right. **a1** High-magnification image from the area indicated with the yellow dashed rectangle in a, showing DCX and BrdU immunostainings. **a2** High-magnification image from the area indicated with the yellow dashed rectangle in **a1**. Newborn neuroblasts (DCX+/BrdU+) in the ventral V-SVZ and NAc. **a3**–**a5**. Left: high-magnification maximum-projection images of newborn neuroblasts (DCX+/BrdU+) in the NAc. Right: orthogonal views. **b** TEM image showing a neuroblast in mitosis. **b1** High-magnification image from the area indicated with the yellow dashed box in **b**. **c** Schematic of the experimental protocol. **d** Left: schematic illustrates the imaged brain area containing the NAc with both core and shell. Right: quantification of newborn neurons in the core and shell of the NAc in adult mice injected with BrdU as depicted in **c** (*n* = 8 mice). **d** Example of newborn cells labeled by BrdU+ and the mature neuronal marker NeuN+. **e** Example of newborn neurons triple-labeled with BrdU, NeuN, and the MSN marker DARPP-32. **f**, **g** Confocal pictures showing BrdU labeling in D1 receptor (EGFP in **f**) or D2 receptor (EGFP in **g**) expressing neurons. Abbreviations: ac, anterior commissure; dSt, dorsal striatum; lv, lateral ventricle; NAc, nucleus accumbens; Sp, septum. Scale bars in µm: 1000 (**a**), 100 (**a1**), 50 (**a2**), 5 (**a3**–**a5**, **e**–**g**), 2 (**b**), 500 nm (**b1**). See Supplementary Fig. 7.

We next investigated whether SVZ-derived neuroblasts migrating to the NAc are generated during adulthood. Eight-week-old adult mice were injected with Bromodeoxyuridine (BrdU) twice a day for 5 days (Fig. 3a). Two days after the last injection, we detected newborn neuroblasts (DCX+/BrdU+) dispersing from the ventral SVZ into the dorsal NAc (Fig. 3a) and some of these cells were actively dividing (DCX+/BrdU+/Ki67+ cells) (Supplementary Fig. 7a, b). The presence of mitotic neuroblasts in the NAc was further corroborated with TEM (Fig. 3b). Of all BrdU+ cells, 40.1 ± 8.9 % were DCX+ (mean ± SD, *n* = 5 mice) and the estimated total number of newborn neuroblasts (DCX+/BrdU+) per NAc was 1254.5 ± 223.9 (mean ± SD, *n* = 8 mice). From these calculations, we infer that ∼250 newborn neuroblasts reach the NAc per day.

An essential question however remains whether V-SVZ-derived neuroblasts mature in the NAc. To address this issue, we employed a BrdU injection protocol that ensures the labeling of a substantial number of newborn cells and the subsequent appraisal of maturation and survival. Specifically, adult mice were injected with BrdU twice a day for 2 weeks and the brains were analyzed 4 weeks after the last injection (Fig. 3c). We found that 7.28 ± 3.18 % of all BrdU+ cells were NeuN+/BrdU+ neurons (mean ± SD, *n* = 6 mice) and the estimated total number of newborn neurons (NeuN+/BrdU+) per NAc was 407.5 ± 155.4 (mean ± SD, *n* = 6 mice). Based on these calculations, we infer that about 29 newborn neurons populate the NAc per day.

A comparable number of NeuN+/BrdU+ mature neurons were found in the core and the shell of the NAc, delineated on the basis of Calbindin staining [30] (Fig. 3d and Supplementary Figs. 7c and  8). Mature NeuN+/BrdU+ neurons were evenly distributed throughout the NAc (Supplementary Fig. 7d) and 91.28 ± 4.92 % (mean ± SD, *n* = 5 mice) co-expressed the MSN marker DARPP-32 (Fig. 3e). Newborn neurons matured both into D1 and D2 receptor- expressing MSNs, as revealed by BrdU labeling in D1-EGFP and D2-EGFP mice [31] (Fig. 3f, g). Notably, the newborn cells are distinct from the Calretinin+/5HT3A+ postnatally born neurons that eventually populate the dorsal striatum, as reported before [18]. The two cell types can be clearly differentiated based on their morphology. Thus, Calretinin+ neurons exhibit small cell bodies with few dendritic branches, whereas newborn MSNs in the NAc have large cell bodies and an elaborate dendritic tree.

**Fig. 4:**
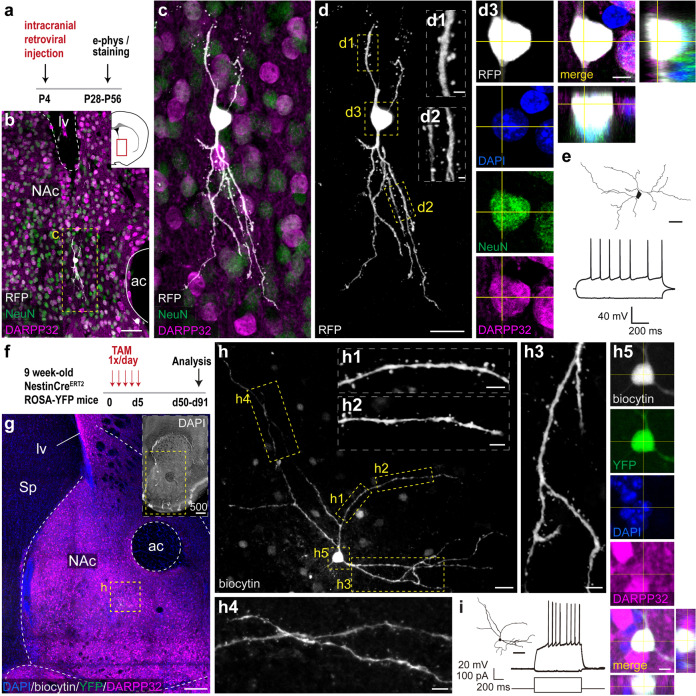
Newborn neurons in the adult NAc become mature MSNs. **a** Experimental schematic: P4-day-old pups were injected with a RFP-expressing retrovirus in the V-SVZ and analyzed 4–8 weeks later. **b** Coronal section of P56 wild-type mouse brain immunostained for RFP, DARPP-32, and NeuN. Inset: schematic of the anatomical region in a coronal section. The red rectangle denotes the area in the picture. **c**, **d** High-magnification images from the area indicated with the yellow dashed rectangle in **b**. **d1**, **d2** Magnifications show segments of a dendrite bearing spines. **d3** High-magnification image of the retrovirus-infected soma, indicating that this cell expresses typical markers for mature MSNs in the NAc (DARPP-32 and NeuN). **e** Top: morphological reconstruction of a biocytin-filled virus-infected neuron in the NAc. Bottom: representative firing patterns of virus-infected neurons recorded in the NAc. **f** Schematic of the experimental protocol. **g** Overview of the NAc immunostained for DAPI, biocytin, DARPP-32, and YFP. Inset: low-magnification image of a coronal brain section of a P130 wild-type mouse immunostained for DAPI. **h** High-magnification image of the yellow dashed rectangle. **h1**–**h4** Enlargement of the areas indicated in the picture, showing dendritic spines. **h5** High-magnification image of the biocytin-filled cell body, showing positive expression for YFP and DARPP-32. **i** Left: morphological reconstruction of biocytin-filled YFP-expressing neuron shown in **h**. Right: representative firing pattern of the YFP-labeled MSN recorded in the NAc. Abbreviations: ac, anterior commissure; NAc, nucleus accumbens; lv, lateral ventricle; Sp, septum. Scale bars in µm: 500 (onset in **g**), 200 (**g**) 50 (**b**, **e**, **i**), 20 (**c**, **d**, **h**), 5 (**d3**, **h1**–**h5**), 1 (**d1**, **d2**). See also Supplementary Fig. 9 and Supplementary Movie 6.

To obtain functional evidence that postnatally generated neurons in the NAc develop a mature phenotype, we performed patch-clamp recording experiments employing two approaches to identify the to-be-patched cells. First, we labeled newborn cells by injecting an red fluorescent protein (RFP)-expressing retrovirus that guarantees the exclusive infection of dividing cells [32] (Fig. 4a) by injecting the virus in the V-SVZ of P4-day-old mice. Mature retrovirally infected cells in the adult NAc showed the typical morphology of MSNs with prominent dendritic spines (Fig. 4b-d). Their identity was further confirmed by immunohistochemistry with NeuN and DARPP-32 markers (Fig. 4d). Infected cells could be easily visualized and patched in acute brain slices, and the firing pattern was assessed. Upon current injection into the cell soma, all patched cells (12 cells from 5 mice) exhibited a firing pattern that is indicative of a mature phenotype (Fig. 4e). Morphological reconstruction of the patched neurons further corroborated the neuronal identity (Fig. 4e).

Second, to further examine electrophysiologically MSNs generated in the adult brain, we employed NestinCre^ERT2^ mice crossed with Rosa-YFP reporter mice, as V-SVZ-derived neural progenitor cells and their progeny are irreversibly labeled upon tamoxifen administration in these mice [33]. We controlled for possible leakiness of the system by analyzing brain sections of transgenic mice that had not received tamoxifen (mice were injected with oil) and confirmed the absence of yellow fluorescent protein (YFP^+^) cells under these conditions (not shown). Nine-week-old transgenic mice were treated with tamoxifen once per day for 5 consecutive days and the brains were analyzed either 1 h or 8 weeks after the last tamoxifen injection (Fig. 4f). In all tamoxifen-treated mice, there was YFP expression in neural progenitor cells lining the walls of the ventricles and in V-SVZ-derived neuroblasts in the RMS (Supplementary Fig. 9a, b). We did not detect YFP-positive cells in the NAc or other brain areas immediately after the last tamoxifen injection (not shown), indicating that only V-SVZ neural precursors were labeled upon tamoxifen injection. Conversely, we found YFP+/DARPP-32+ neurons scattered throughout the NAc in mice killed 8 weeks after tamoxifen administration (85% of YFP+ cells in the NAc were positive for DARPP-32) (Fig. 4g, h and Supplementary Fig. 9a, c, d). Thus, by this time point most YFP+ cells in the NAc developed a MSN phenotype. Based on morphology, we infer that the remainder of YFP+ cells developed into other cell types such as interneurons and oligodendrocytes (data not shown). Electrophysiological recordings were performed in brain slices from mice killed 6–13 weeks after tamoxifen administration. Upon current injection into the cell body, patched cells showed a firing pattern comparable to what has been reported for prenatally generated MSNs [34] (Fig. 4i and Supplementary Fig. 9e). Patched cells exhibited a slow depolarizing ramp and instant inward rectification when hyperpolarized from resting membrane potential by current injection (Fig. 4i and Supplementary Fig. 9e). Biocytin filling into patched neurons permitted subsequent reconstruction to establish cell morphology and location after tissue fixation. The cell identity of YFP+ as MSNs was further confirmed based on the presence of characteristic dendritic spines and DARPP-32 expression (Fig. 4h, Supplementary Fig. 9d, and Supplementary Movies 6 and 7). Morphological reconstruction of biocytin-labeled cells in 300 µm-thick coronal sections revealed cellular projections within the NAc (Supplementary Movies 6 and 7). Altogether, our results demonstrate that new MSNs are born in the adult V-SVZ, mature, and integrate in the NAc.

### Inflammatory and neuropathic pain induce neurogenesis in the NAc

**Fig. 5:**
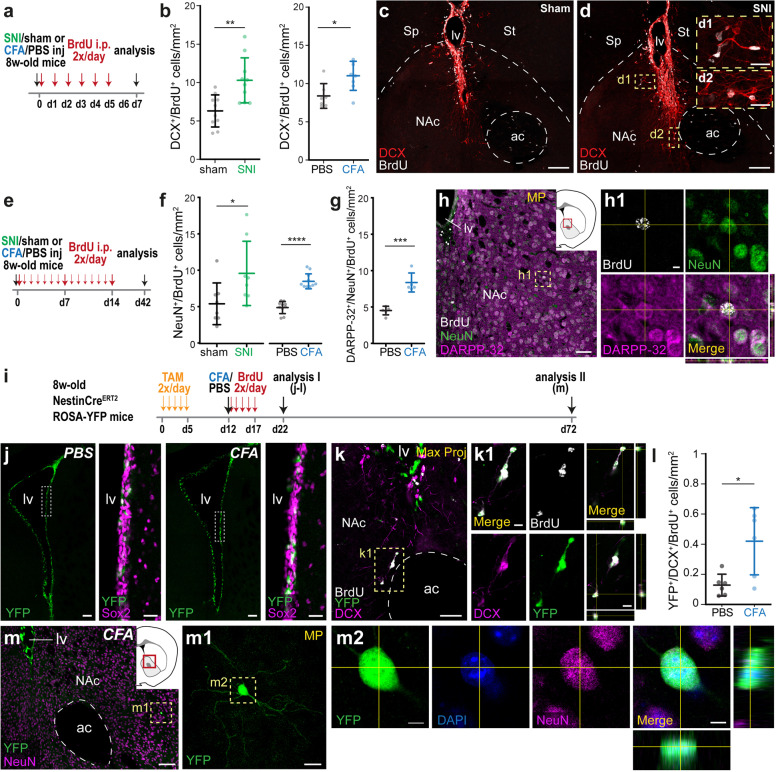
Enhanced neurogenesis in the NAc following inflammatory and neuropathic pain. **a** Schematic of the experimental protocol. **b** Quantification of newborn neuroblasts in the NAc 7 days after sham or SNI surgery (left) (unpaired *t-*test *p = 0.0026, *n* = 10 mice per group) or after PBS or CFA paw injection (right) (unpaired *t-*test *p = 0.0131, *n* = 7 and 8 mice per group, respectively). **c**, **d** Representative pictures of coronal brain sections from mice subjected to either sham (**c**) or SNI surgery (**d**), stained with antibodies against BrdU (white) and DCX (red). **d1**, **d2** High-magnification images of the dashed yellow rectangles are shown on the right. **e** Experimental schematic to test the effect of chronic pain in the inflammatory model (CFA/PBS injection) or in the neuropathic pain model (SNI/sham) in the NAc after 6 weeks. **f** Quantification of mature postnatally born neurons (NeuN+/BrdU+) in the NAc, 4 weeks after the last BrdU injection (left: unpaired *t-*test *p = 0.041, *n* = 8 mice per group for sham/SNI; right: unpaired *t-*test ****p < 0.0001, *n* = 10 mice per group for PBS/CFA injections). **g** Quantification of mature postnatally born neurons (NeuN+/BrdU+) in the NAc counterstained with the MSNs marker DARPP-32, 6 weeks after CFA or PBS injection (unpaired *t-*test ***p = 0.0003, *n* = 5 mice per group). **h** Representative picture of a coronal brain section from a CFA-treated mouse showing examples of BrdU+ (gray)/ NeuN+ (green)/ DARPP-32+ (magenta) neurons in the NAc. **h1** Higher magnification of the dashed yellow rectangle for each channel is shown on the right. The inset indicates the imaged brain area (dashed yellow rectangle). **i** Experimental schematic to test the origin of NAc adult-born neurons after inflammatory pain. **j** Representative pictures of coronal sections comprising the lateral ventricles showing YFP-labeled neural stem cells in the V-SVZ of control (left) and CFA-treated mice (right). Pictures on the right are enlargements of the indicated dashed yellow rectangles showing YFP-labeled cells (green) positive for Sox2 (magenta). **k** Confocal picture showing newborn (BrdU in white) neuroblasts (DCX in magenta) positive for YFP (green) with migratory appearance in the NAc of a CFA-treated mouse. **k1** Enlarged pictures of the boxed area in **k** for each channel. **l** Quantification of (DCX+/BrdU+) newborn neuroblasts in the NAc positive for YFP in control (PBS) or pain (CFA)-treated animals (*t*-test *p = 0.035, *n* = 6 mice per group). **m** Representative image of a coronal brain section from a CFA-treated mouse stained with antibodies against YFP (green) and NeuN (magenta), showing newborn neurons in the NAc 72 days after the first tamoxifen injection. **m1** Enlarged picture of the dashed yellow rectangle in **m** for the YFP+ cell. **m2** Enlarged pictures of the dashed yellow rectangle in **m** for each channel, including DAPI (blue). Scale bars in µm: 100 (**c**, **d**, **m**, **j**), 50 (**h**, **k**), 25 (**d1**, **d2**, **m1**), 10 (**k1**), 5 (**m2**). Abbreviations: ac, anterior commissure; lv, lateral ventricle; NAc, nucleus accumbens; Sp, septum; St, striatum. See also Supplementary Figs. 10 and 11.

There is increasing evidence in humans and rodents that the NAc is involved in modulating pain. Thus, the NAc becomes active during pain, in line with its general role in responding to salient stimuli, regardless whether these are positive or negative [35–40]. To study whether persistent flow of nociceptive activity into the brain modifies neurogenesis in the NAc, we employed mice subjected to a model of chronic neuropathic pain resulting from traumatic nerve injury, namely the spared nerve injury (SNI) model (Fig. 5a). Mice were intraperitoneally (i.p.) injected with BrdU twice a day for 5 days, starting on the same day as the SNI. Two days after the last BrdU injection, newborn immature neurons (DCX+/BrdU+) were counted in the NAc (Fig. 5a). We found increased neurogenesis in the NAc of mice that underwent SNI as compared with sham-operated mice (Fig. 5b-d). To evaluate whether an increase in neurogenesis can be triggered in other pain models, we employed a model of inflammatory pain induced by plantar injection of Complete Freund’s adjuvant (CFA) in one hind paw (Fig. 5a). Control mice were injected with phosphate-buffered saline (PBS). BrdU was administered twice a day for 5 days, starting on the day of the PBS/CFA injection. Two days after the last BrdU dose, newborn immature neurons (DCX+/BrdU+) were counted in the NAc (Fig. 5a). There was a significantly higher number of newborn neuroblasts (DCX+/BrdU+) in mice with inflammatory pain in comparison with controls (Fig. 5b).

To study whether pain-modulated neurogenesis in the NAc leads to an altered number of mature MSNs, mice were i.p. injected with BrdU twice a day for 2 weeks subsequent to sham or SNI surgeries on the one hand (BrdU treatment starting on the second day after surgery), or PBS or CFA injection on the other (BrdU treatment starting on the same day) (Fig. 5e). Mature newborn neurons (NeuN+/BrdU+) in the NAc were quantified 4 weeks after the last BrdU injection (Fig. 5e). The number of mature neurons in the NAc of mice with SNI surgery or inflammatory pain was ∼1.75-fold higher compared with that in the respective control group (Fig. 5f). Notably, the pain-induced augmentation of newly generated neurons was consistent and comparable in both models. Furthermore, DARPP-32 labeling revealed that most of the newborn NeuN+/BrdU+ neurons were MSNs (91.28 ± 4.92% and 94.29 ± 4.86%, mean ± SD, *n* = 5 mice per group, PBS and CFA-treated groups, respectively) (Fig. 5g, h). We quantified the increase in the density of NeuN+/BrdU+ neurons in the NAc core and shell of CFA-treated mice, and found no significant differences between the two areas (1.90 ± 0.25-fold and 1.78 ± 0.39-fold, mean ± SEM for core and shell, respectively, *t*-test *p* = 0.802).

As neuropathy or inflammation were induced unilaterally in both pain models, we investigated whether the increase in newborn neurons in the NAc was confined to one hemisphere, but found a similar increase in the number of newborn neurons in both hemispheres (Supplementary Fig. 10a, b). Pain-induced augmented neurogenesis appears to be restricted to specific circuits, as there was no difference in the number of SVZ-derived newborn cells in the OB of mice from the 2 treatment groups (Supplementary Fig. 10a, b).

### Newborn neurons generated in pathological pain states originate in the SVZ

Although our data clearly demonstrated that the postnatal and adult SVZ generate neurons that target the NAc, one remaining question was whether the SVZ is also the source for the above-described augmented neurogenesis after pathological pain. To investigate this issue, we used NestinCre^ERT2^ Rosa-YFP reporter mice, as before. Adult transgenic mice were treated with tamoxifen twice per day for 5 consecutive days. After a 1-week-long chase period, we performed PBS or CFA injections together with BrdU administration twice per day for 5 consecutive days, and analyzed 10 days later the distribution of YFP-labeled newborn cells (analysis time point I) (Fig. 5i). In all tamoxifen-treated mice, independently of the treatment, there was YFP expression in stem cells lining the walls of the ventricles (Fig. 5j). Importantly, we detected YFP+/DCX+/BrdU+ cells in the NAc, and the number of triple-positive cells was significantly increased in mice with inflammatory pain (Fig. 5k, l). We hence conclude that adult-generated neurons in response to pain stimuli in the NAc derived from the SVZ. Finally, we further confirmed that, also in this model, newborn neuroblasts generated after pain survived and matured into neurons in the NAc (analysis time point II). Indeed, 60 days after CFA injection, YFP+ cells in the NAc were positive for NeuN and DARPP-32, and exhibited neuronal morphology (Fig. 5m and Supplementary Fig. 11a-c).

## Discussion

In this study we provide evidence for adult neurogenesis in the NAc. Specifically, we report NAc-specific migration of V-SVZ-generated neuroblasts in close vicinity to long RG-like fibers, and we demonstrate that projection neurons—MSNs—are generated in adult mice under physiological conditions. We detected newborn postnatal and adult MSNs using three in vivo approaches: BrdU administration, retroviral injections, and labeling of stem cells and their progeny in adult conditional transgenic mice. Employing two different animal models, we also show that adult NAc neurogenesis is modulated by pain-derived patho-physiological responses, indicating that environmental factors alter the final number of newly generated MSNs in the NAc.

The direct contact of the dorsal NAc with the anterior–ventral lateral ventricle and the presence of immature neuroblasts with migratory morphology dispersing from the avV-SVZ suggested that newborn neurons in the NAc derive from the V-SVZ. This was indeed the case, as we demonstrated using two approaches: (a) live-cell imaging on ex vivo brain sections from adult mice and (b) inducible genetic fate-mapping experiments where only V-SVZ-stem cells (and not cells in the NAc) were fluorescently labeled upon a tamoxifen pulse. A number of reports have previously identified several substrates that are used by neuroblasts while migrating in the adult brain. For instance, V-SVZ-derived neuroblasts move along other neuroblasts [41], blood vessels [42–44], serotonergic axons [28] and astrocytic processes [41, 45–47], which serve as scaffolds providing mechanical and/or biochemical support. During embryonic development, neuronal precursors generated in the VZ migrate following RG processes that extend from the VZ to the pia [3, 48]. RG cells cover the surface of the ventricle in the embryo brain, but they disappear from the dorsal ventricular wall shortly after birth [49, 50], giving place to adult NSCs. However, only in the adult NAc some fibers remain that display RG-like NSC hallmarks such as expression of astrocytic and NSC markers like GFAP, Vimentin, and Nestin [19]. In addition, these RG-like cells extend long processes (up to 1 mm in some cases) and reach blood vessels in the NAc. The intimate association between neuroblasts and astrocytic processes strongly suggests that adult-born neuroblasts migrate along processes from RG-like cells before differentiating into MSNs in the NAc. This is reminiscent of developmental migration of immature MSNs in the LGE (Supplementary Fig. 5e).

Previous studies have shown that embryonic RG cells from the LGE produce the majority of striatal MSNs [21–23] and also a large number of postnatal NSCs that populate the avV-SVZ [8, 9]. LGE-derived adult NSCs, in turn, generate certain types of interneurons that migrate mainly to the OB [9]. Interestingly, prenatally generated striatal MSNs and adult NSCs from the ventral V-SVZ share a common progenitor [9]. Indeed, the expression of specific transcription factors that regulate neurogenesis, such as SP8 and SP9, overlaps between embryonic MSN progenitors and adult OB neuron progenitors [51–54]. Given the anatomical location and embryonic origin of NSCs residing in the adult ventral V-SVZ, one can presume that these cells have the capacity of producing the postnatally born MSNs detected in this study.

The presence of newborn neurons in rodent postnatal and adult brain regions other than the OB and the hippocampal dentate gyrus (DG) has been controversially discussed over the years [55]. By now, based on numerous studies, the following scenario emerged: brain areas that were considered virtually non-neurogenic in rodents—indeed they harbor only few newborn neurons compared with what was reported for the OB and DG—may be the final destination of postnatally born neurons in humans. This is, for instance, the case for the neocortex in infants [56, 57], for the amygdala during adolescence [58], and possibly for the striatum [59] and hypothalamus [60] in adults. Over the last years a number of studies have emerged in favor [61–63] but also against [64–66] the existence of adult human hippocampal neurogenesis. Thus, the issue whether and to which extent neurogenesis in the adult human hippocampus occurs has not been conclusively resolved as of now. The human V-SVZ contains NSCs, although this germinal activity sharply declines during the first postnatal years [56, 57, 67]. In contrast to the case of rodents, no immature neurons migrating to the OB were detected in the adult human brain [57, 68] despite the presence of neuroblasts in the adult human V-SVZ [68, 69]. Whether V-SVZ-derived neuroblasts migrate to the NAc and differentiate into MSNs in the human postnatal brain, either at young or adult ages, remains to be explored.

A central question in the field of adult neurogenesis revolves around the functional significance of newly born neurons. By now, a substantial number of studies have revealed that adult neurogenesis supports tissue homeostasis and contributes to specific aspects of the biological functions normally executed by cells in the brain area where newborn neurons integrate. For example, hippocampal and OB neurogenesis are involved in spatial memory and odor processing, respectively (reviewed in ref. [70]). Consistently, the extent of adult neurogenesis is dynamically regulated by different stimuli that act in a region-specific manner, e.g., the hippocampus or olfactory-dependent learning promotes neurogenesis in the DG or OB, respectively [71, 72]. Although the function of the NAc has been classically linked to motivation and addiction [73], recent studies revealed that the NAc is also involved in chronic pain processing [74–76]. Our results showed that pathological pain increases the number of new neurons in the NAc, thus, it is tempting to speculate that newly generated MSNs might participate in networks that support exacerbated responses to nociceptive stimuli developed during chronic pain conditions. Unfortunately, current methods used to ablate neurogenesis, be that AraC infusion [77, 78], X-irradiation [79], mouse genetic models [51, 53, 80–82], or viral injections [83] would not only affect NAc neurogenesis, but also other major neurogenic niches. Hence, the lack of available tools to manipulate neurogenesis specifically in the NAc precluded us from further probing the functional significance of NAc neurogenesis. Identification of specific genes expressed in adult-generated MSN progenitors would hopefully allow us to tackle this issue in the future.

Although methodological differences (i.e., BrdU injection protocols) do not allow us to establish precise comparisons between NAc neurogenesis and other neurogenic areas in the adult mouse brain, we estimate that the generation of newborn neurons in the NAc is comparable to that in the amygdala [84], but one and three orders of magnitude lower than that in the DG [85, 86] and OB [13, 87], respectively. The functional impact of newborn neurons cannot be inferred based on their number, given the evidence that the activation of single neurons can translate into a defined behavior [88–92]. Neurogenesis in the striatum has been most frequently investigated in the context of damaged brain, including vascular insult and selective loss of substantia nigra dopaminergic neurons [93, 94]. From these studies, it can be inferred that various types of insults augment neurogenesis in the striatum. Upon maturation, some of the newborn cells were shown to develop a Calretinin+ or DARPP-32+ phenotype [95, 96]. Neurogenesis in the striatum has also been reported before under physiological conditions in different species. However, only GABAergic interneurons have been identified so far and the number thereof was quite modest [16, 59, 97–99]. Considering these findings, the significance of our study is twofold. First, we demonstrate that newborn MSNs containing D1-type and D2-type dopamine receptors are produced in the adult healthy brain. Second, by employing pain paradigms, we provide evidence that pathological conditions that are not associated with neuronal death modulate neurogenesis of MSNs. It remains to be seen whether other physiological or pathological stimuli, such as pregnancy and drug addiction [100, 101] known to influence neurogenesis in the V-SVZ promote the generation of MSNs in the NAc. In sum, we identified the NAc as yet another brain area in which postnatal and adult neurogenesis supports a form of environmental-plasticity akin to what has been reported for the DG and the OB.

## Experimental procedures

### Animals

All animal procedures were in accordance with the DKFZ Animal Care guidelines and the local governing body (Regierungspräsidium Karlsruhe, Germany). We used wild-type C57BL/6J mice, 5HT3A-EGFP mice [18], D1 and D2 EGFP mice [31], and NestinCre^ERT2^/R26R-YFP mice [33]. In all experiments, we used both male and female mice indistinctively. Unless stated otherwise, mice were deeply anesthetized with a combination of xylazine (8%; 20 mg/ml) and ketamine (20%; 50 mg/ml) solutions injected i.p. Cre^ERT2^ activation: Tamoxifen (Sigma-Aldrich) was first dissolved in ethanol (65 mg/ml) and diluted in corn oil to a final concentration of 10 mg/ml. Mice were injected i.p. with 1 mg of tamoxifen twice a day for 5 consecutive days. BrdU injections were performed i.p., 50 mg/kg for adult mice and 30 mg/kg for pups, as described before [102, 103].

### Time-lapse video microscopy

Brains were removed from anesthetized P32–42 5HT3A-EGFP mice. Two hundred-micrometer-thick sagittal slices were processed as described [28]. Briefly, sagittal acute sections were sliced in a 4 °C solution containing 125 mM NaCl, 25 mM NaHCO_3_, 1.25 mM Na, H_2_PO_4_, 2.5 mM KCl, 2 mM CaCl_2_, 1 mM MgCl_2_, 25 mM glucose, and bubbled with 95% O_2_/5% CO_2_ at pH 7.4. Imaging was performed on a TCS SP5 microscope (Leica Biosystems, Germany) equipped with a ×20(1 NA) water-immersion objective. Movies were made from three-dimensional stacks acquired sequentially every 5 min for 4 h at the level represented in Fig. 1d, approximately. Stack maximum intensity projections were subsequently aligned in FIJI software and the Manual Tracking plugin was used to track neuroblasts [104], obtained from six independent experiments.

### Immunostainings

Animals were deeply anesthetized and perfused with 4% paraformaldehyde (PFA). Brains were dissected and post-fixed in 4% PFA overnight at 4 °C. Fifty-micrometer-thick sections were prepared using a Leica VT100S vibratome (Leica Microsystems GmbH). Slices were blocked in 0.2–1% Triton and 3% bovine serum albumin, and incubated overnight with primary antibodies at 4 °C followed by incubation with secondary antibodies at room temperature. For BrdU stainings, slices were preincubated with 1 N HCl at 45 °C and neutralized with 10 mM Tris (pH 8.5) at room temperature. Brain sections were stained for the following: EGFP/YFP (rabbit, Invitrogen, Cat# A-6455, 1 : 1000), BrdU (mouse, BD, Cat# 347580, 1 : 1000), NeuN (rabbit and mouse, Millipore, Cat# MAB377, 1 : 1000; chicken, Synaptic System, Cat# 266 006, 1 : 250), DARPP-32 (rabbit, Santa Cruz, Cat# ab40801, 1 : 300), Calbindin (rabbit, Swant, Cat# 300, 1 : 3000), DCX (goat, Santa Cruz, Cat# sc-8066, 1 : 500), Iba1 (rabbit, Abcam, Cat# EPR16588, 1 : 500), Olig2 (rabbit, Invitrogen, Cat# PA5–85734, 1 : 500), PSA-NCAM (mouse, Thermo Fisher Scientific, Cat# 14–9118–80, 1 : 250), Collagen IV (rabbit, Abcam, Cat# ab19808, 1 : 500), Nestin (chicken, Novus Biological, Cat# NB100–1604, 1 : 250), GFAP (rabbit, DAKO, Cat# GA52461–2, 1 : 500), Vimentin (goat, Santa Cruz, Cat# sc-7557, 1 : 500), Sox2 (Santa Cruz, Cat# sc-17320, 1 : 500), Ki67 (rabbit, Abcam, Cat# ab15580, 1 : 250). For nuclear staining, we used DAPI (Invitrogen).

### Quantification of the distance between glial fibers and neuroblasts

We used the approach previously employed [28, 105]. In brief, 50 μm-thick coronal sections containing the NAc from four adult brains (P65) were analyzed. Images were acquired with a confocal microscope (Zeiss) in z-stacks of 8 µm in depth with a resolution of 1024 × 1024 pixels from five sections per brain. Using the FIJI software [104], the distance between each DCX+ neuroblast cell body and the closest glial process was determined. Using the same image, a grid (grid type: lines) was placed with a random offset and an area per point of 2000 µm [2], to generate random-distributed points with a density similar or higher to the one of the neuroblasts in the area. The minimal distance between the intersection point of the grid lines and the closest glial process was quantified per field in the whole z-stack. Thus, each individual dot in Fig. 2c represents the minimal distance calculated between DCX+ neuroblasts and the closest glial fiber (*n* = 469 measurements), and between the grid line intersection points and the closest glial fiber (*n* = 600 measurements). As a control, similar estimations were performed with a different grid with smaller areas per point (1000 and 500 µm^2^), and identical results were obtained.

To further analyze the interaction of glial fibers and migrating neuroblasts, we studied the distribution of DCX+ neuroblasts and GFAP+ glial fibers dispersing from the avV-SVZ towards the NAc. Considering the closest point in the V-SVZ as origin, we obtained a 2D vector representing the position of each DCX+ neuroblast (containing the distance and the angle between 0° and 180°). The same procedure was followed for the nearest GFAP+ glial fiber. In Fig. 2d, each dot represents an individual DCX+ cell or the nearest GFAP+ glial fiber (*n* = 755 measurements for each) and contours represent the average for each group relative to the origin in the V-SVZ taking bins of 7.5°. Graphs and analyses were performed with Sigmaplot for Windows (14.0) and GraphPad Prism.

### Quantification of the total number of newborn neurons in the NAc

Cells were counted in z-stack images from 50 µm-thick sections stained with DCX/BrdU, NeuN/BrdU, and DARPP-32/BrdU markers. Eight to 12 representative images across evenly spaced and randomly sampled sections from the total number of NAc-containing sections (~25 on average) were collected for quantification at each age. Optical sections of 8 µm-thick were analyzed for cell countings. The NAc was initially identified using Calbindin and DAPI (4′,6-diamidino-2-phenylindole) labeling to establish the NAc boundaries (dorsal: ventral V-SVZ; ventral: ventral pallidum and olfactory tubercle; medial: Calleja Islands; lateral: lateral stripe of the striatum). Calbindin labeling allowed to determine the boundaries between NAc core and shell [30]. Each mouse represents *n* = 1. Counts for DCX+/BrdU+ were performed by three different investigators for reproducibility. For newborn mature neurons (NeuN+/BrdU+ and DARPP-32+/BrdU+), counts were repeated by two separate investigators.

### Transmission electron microscopy

Adult mice (P56) were deeply anesthetized and perfused with 0.9% saline followed by 2% PFA and 2.5% glutaraldehyde (EMS, Hatfield, PA, USA) in 0.1 M phosphate buffer (PB). Brains were dissected and post-fixed overnight at 4 °C in the same fixative solution and, subsequently, 200 µm transversal sections were prepared using a Leica VT1200S vibratome (Leica Microsystems GmbH, Heidelberg, Germany). Slices were further post-fixed in 2% osmium tetroxide in 0.1 M PB for 1.5 h at room temperature, washed in deionized water, and partially dehydrated in 70% ethanol. Samples were then incubated in 2% uranyl acetate in 70% ethanol in the dark for 2.5 h at 4 °C. Brain slices were further dehydrated in ethanol followed by propylene oxide and infiltrated overnight in Durcupan ACM epoxy resin (Fluka, Sigma-Aldrich, St. Louis, USA). The following day, fresh resin was added and the samples were cured for 72 h at 70 °C. Following resin hardening, ultrathin sections (70–80 nm) were obtained with a diamond knife using a Ultracut UC7 ultramicrotome (Leica), stained with lead citrate and examined under a FEI Tecnai G^2^ Spirit TEM at 80 kV (FEI Europe, Eindhoven, The Netherlands) equipped with a Morada CCD digital camera (Olympus Soft Image Solutions GmbH, Münster, Germany).

### Viral production and injection

The packaging cell line HEK293 was co-transfected with the viral backbone vector (a replication-deficient Moloney murine leukemia retrovirus expressing RFP driven by the CAG promoter) plus the helper plasmids, and the retroviral particles were purified by ultracentrifugation. The cell lines were authenticated and tested for mycoplasma contamination. The concentrated viral solutions (10^6^–10^8^ cfu/ml) were titrated and stored at −80 °C until usage. One to 2 µl of the viral solution (depending on the viral titer) were injected with a glass capillary into the V-SVZ of anesthetized pups at postnatal day 4. The following coordinates from Bregma were used: 0.6 anterior, 1.2 lateral, 1.5 ventral. The animals were placed on a heating pad while anesthetized and returned to their home cages with the mother after the surgery.

### Slice preparation and whole-cell recordings

Mice were deeply anesthetized with isoflurane, followed by transcardially perfusion with 30 ml sucrose solution containing (in mM) 212 sucrose, 26 NaHCO_3_, 1.25 NaH_2_PO_4_, 3 KCl, 7 MgCl_2_, 10 glucose, and 0.2 CaCl_2_, cooled to 4 °C, and oxygenated with carbogen gas (95% O_2_/5% CO_2_, pH 7.4). Acute coronal sections (300 μm), including NAc shell, were prepared with a vibratome (Slicer HR2, Sigmann Elektronik, Germany) and the tissue was incubated in sucrose solution at 35 °C room temperature for 30 min and transferred to oxygenated extracellular solution containing (in mM) 125 NaCl, 25 NaHCO_3_, 1.25 NaH_2_PO_4_, 2.5 KCl, 2 CaCl_2_, 1 MgCl_2_, and 25 glucose at room temperature (22–25 °C). Recording pipettes, pulled from borosilicate capillaries with the tip resistance of 4–7 MΩ and were filled with a low Cl^−^ potassium-based solution containing (in mM): 130 K-gluconate, 10 Na-gluconate, 10 Hepes, 10 phosphocreatine, 4 NaCl, 4 Mg-ATP, and 0.3 GTP, pH adjusted to 7.2 with KOH.

Liquid junction potentials were not corrected. Series resistance was maximally compensated and continuously monitored during the recordings. Cells were discarded if no “Giga seal” was initially obtained or series resistance changed more than 20% or was higher than 25 MΩ. YFP-expressing cells could be visualized by using epifluorescence and DIC optics. Action potential generation was tested by keeping the cells in whole-cell current-clamp mode at resting potential and applying square pulses (of 1 s duration), starting from −100 pA in 20 pA steps until maximal firing frequency was reached. Input resistance was estimated by averaging the 30 traces of passive membrane potential change in response to −10 pA injection. All recordings were performed using HEKA PatchMaster EPC 10 amplifier. All signals were filtered at 3 kHz and sampled at 20 kHz.

### Cell identification and morphological reconstruction

For subsequent morphological characterization of the patched cells, biocytin (10 mg/ml; Sigma) was added to the intracellular solution. Acute slices with biocytin-filled cells in the NAc were fixed overnight in 4% PFA, followed by extensive washing with PBS and quenched in 1% H_2_O_2_, followed by incubation with avidin–biotin–horseradish peroxidase complex (Elite ABC, Vector Laboratories). The immunoperoxidase reaction was developed using 3,3’-diaminbenzidine (Sigma) as chromogen. Three-dimensional neuronal reconstructions were performed using the Neurolucida software package (MBF Bioscience).

### Inflammatory and neuropathic pain models

For induction of paw inflammation, 20 μl of CFA (Sigma-Aldrich) was injected under isoflurane anesthesia subcutaneously into the plantar surface of the right hind paw, as described previously [106]. The control group was injected with sterile PBS and did not develop inflammation. In this pain model, BrdU administration started immediately after the injection.

The surgical procedure for the SNI model of neuropathic pain was performed under isoflurane general anesthesia, as described previously [107]. Briefly, the common peroneal and tibial branches of the right sciatic nerve were ligated and cut. A 1–2 mm portion of the nerve was removed. Sham surgery refers to the same surgical operation without injury to the nerves. In this pain model BrdU administration started 2 days after the surgery, to exclude the pain effect of the surgery itself.

### Image acquisition

To quantify the number of newborn cells in the NAc, tile images containing the avV-SVZ and the anterior commissure were acquired with a Zeiss (LSM 700) and with a Leica SP8 confocal microscope. We selected 7–12 coronal slices per mouse, spanning the entire NAc. Positive cells for the indicated cell marker per slice were quantified and the mean number from all slices was calculated per mouse.

### Statistical analysis

Statistical analyses were performed with GraphPad Prism software. Sample size was not predicted, no data was excluded, and the experimentators were not blinded. Animals were randomly selected for each experimental group. The datasets were first tested for a normal distribution and equal variance. We used two-tailed unpaired *t-*test and one-way analysis of variance repeated measures (followed by Bonferroni’s multiple comparisons test) to compare normally distributed data, or Mann–Whitney test, Kruskal–Wallis test (followed by Dunn’s multiple comparisons test), and Friedman test (followed by Dunn’s multiple comparisons test) for datasets that did not pass the normality test. Data are presented as mean ± SD unless otherwise indicated.

## Supplementary information

Supplementary Figure Legends

Suppl Fig 1

Suppl Fig 2

Suppl Fig 3

Suppl Fig 4

Suppl Fig 5

Suppl Fig 6

Suppl Fig 7

Suppl Fig 8

Suppl Fig 9

Suppl Fig 10

Suppl Fig 11

Supplementary Movie 1

Supplementary Movie 2

Supplementary Movie 3

Supplementary Movie 4

Supplementary Movie 5

Supplementary Movie 6

Supplementary Movie 7
