# Correction: Neurogenesis of medium spiny neurons in the nucleus accumbens continues into adulthood and is enhanced by pathological pain

**DOI:** 10.1038/s41380-021-01177-z

**Published:** 2021-06-07

**Authors:** Diego García-González, Ionut Dumitru, Annalisa Zuccotti, Ting-Yun Yen, Vicente Herranz-Pérez, Linette Liqi Tan, Angela Neitz, José Manuel García-Verdugo, Rohini Kuner, Julieta Alfonso, Hannah Monyer

**Affiliations:** 1 Department of Clinical Neurobiology, University Hospital Heidelberg and German Cancer Research Center, Heidelberg, Germany; 2 Taiwan International Graduate Program in Molecular Medicine, National Yang-Ming University and Academia Sinica, Taipei, Taiwan; 3 Laboratory of Comparative Neurobiology, Cavanilles Institute, University of Valencia, CIBERNED, Valencia, Spain; 4 Predepartamental Unit of Medicine, Faculty of Health Sciences, Universitat Jaume I, Castelló de la Plana, Spain; 5 Institute of Pharmacology, Heidelberg University, Heidelberg, Germany; 6 Present Address: Biotech Research and Innovation Centre (BRIC), Faculty of Health, University of Copenhagen, Copenhagen, Denmark; 7 Present Address: Department of Cell and Molecular Biology, Karolinska Institute, Stockholm, Sweden

**Keywords:** Neuroscience, Stem cells, Depression

Correction to: *Mol Psychiatry*

10.1038/s41380-020-0823-4

The article “Neurogenesis of medium spiny neurons in the nucleus accumbens continues into adulthood and is enhanced by pathological pain”, written by Diego García-González, Ionut Dumitru, Annalisa Zuccotti, Ting-Yun Yen, Vicente Herranz-Pérez, Linette Liqi Tan, Angela Neitz, José Manuel García-Verdugo, Rohini Kuner, Julieta Alfonso & Hannah Monyer, was originally published electronically on the publisher’s internet portal on 1 July 2020 without open access. With the author(s)’ decision to opt for Open Choice the copyright of the article changed on 11 May 2021 to © The Author(s) 2021 and the article is forthwith distributed under a Creative Commons Attribution 4.0 International License, which permits use, sharing, adaptation, distribution and reproduction in any medium or format, as long as you give appropriate credit to the original author(s) and the source, provide a link to the Creative Commons licence, and indicate if changes were made. The images or other third party material in this article are included in the article’s Creative Commons licence, unless indicated otherwise in a credit line to the material. If material is not included in the article’s Creative Commons licence and your intended use is not permitted by statutory regulation or exceeds the permitted use, you will need to obtain permission directly from the copyright holder. To view a copy of this licence, visit http://creativecommons.org/licenses/by/4.0. Open access funding enabled and organized by Projekt DEAL.

