# Supplementary material for: Neurogenesis of medium spiny neurons in the nucleus accumbens continues into adulthood and is enhanced by pathological pain
Source: Mol Psychiatry. 2020 Jul 1;26(9):4616–32. doi: 10.1038/s41380-020-0823-4 (PMC8589654; doi:10.1038/s41380-020-0823-4)
Supplement: Supplementary file 1 — Supplementary Figure Legends [file 41380_2020_823_MOESM1_ESM.docx]

**Supplementary Figure Legends**

***Supplementary Figure 1. Neuroblast identity of DCX+ and PSA-NCAM+ cells in the adult NAc.***

**a**. Schematic depicting the avV-SVZ in a V-SVZ whole-mount preparation. **b**. Confocal image of coronal brain section from a P70 wild-type mouse. DCX+ neuroblasts do not co-express the oligodendroglial marker Olig2. **c**. High-magnification maximum-projection image of a coronal brain section from a P70 wild-type mouse. PSA-NCAM+ neuroblasts do not express the microglial marker Iba1. **c1**. High-magnification confocal image and orthogonal views of the dashed boxed rectangle in b. Scale bars in µm: 25 (b, c), 5 (c1).

***Supplementary Figure 2. V-SVZ-derived Neuroblasts migrate into the NAc.***

**a.** Sagittal brain section of a P120 wild-type mouse, immunostained for Ki67. **a1** and **a2** are magnifications of the inset marked in dashed yellow rectangles in a and a1, respectively. Note that at P120 fewer DCX+ neuroblasts are detected in the dorsal NAc than at P40. **b.** Sagittal brain section of a P33 5HT3A-EGFP mouse, stained with DAPI. **b1** is a magnification of the inset marked in a dashed yellow rectangle in b. **b1**-**b3**. 5HT3A+/DCX+-expressing neuroblasts migrate into the NAc from the avV-SVZ. Note that the sagittal section shown in b was first imaged in time-lapse video recordings. Post-hoc immunohistochemical analysis was performed to confirm DCX expression in V-SVZ-derived migrating 5HT3A+-neuroblasts. Abbreviations: 3v – third ventricle; ac – anterior commissure; lv – lateral ventricle; NAc – nucleus accumbens; OB - olfactory bulb; RMS - rostral migratory stream; avV-SVZ – anterior-ventral subventricular zone. Scale bar in µm: 500 (b), 50 (a1, a2, b1), 20 (b2), 10 (b3).

***Supplementary Figure 3. Long radial glia-like fibers in the adult NAc.***

**a**. Coronal brain section of a P70 wild-type mouse. Vimentin+/GFAP+ glial fibers extend from the ventral V-SVZ selectively within the NAc. **b**. RG-like processes originating in the adult avV-SVZ exhibit a NAc-specific preference. **c**. Maximum projection of a coronal brain section of a P70 wild-type mouse containing the ventral V-SVZ. **d**. Enlarged pictures of the dashed yellow rectangle in c. Orthogonal views of each separate channel showing a DAPI/Vimentin+/Ki67+ cell. **e**. Enlarged pictures of the dashed yellow rectangle in d. The white arrow indicates plane-by-plane the DAPI/Vimentin+/Ki67+ cell. Abbreviations: ac – anterior commissure; dSt – dorsal striatum; lv – lateral ventricle; NAc – nucleus accumbens; Sp – Septum. Scale bar in µm: 500 (a), 50 (b, c), 20 (d), 5 (e).

***Supplementary Figure 4. Fibers from radial glia-like cells in the avV-SVZ display long basal processes with end feet on blood vessels in the NAc.***

**a.** Sagittal section of the V-SVZ-NAc from a P70 wild-type mouse. **b**. Enlarged picture of the dashed yellow rectangle showing Vimentin+/GFAP+ glial fibers in the V-SVZ and NAc. **c**, **d**, **e**. Enlarged pictures of the dashed yellow rectangles indicated in b. **c**. A blood vessel outside the NAc does not receive long basal end feet processes from Vimentin+ radial glia-like cells. **d**, **e**. A collagen IV+ blood vessels in the NAc receives numerous end feet processes from Vimentin+ radial glia-like cells and is wrapped by local astrocytes. Abbreviations: ac – anterior commissure; lv – lateral ventricle; NAc – nucleus accumbens. Scale bar in µm: 500 (a), 100 (b), 50 (c, d), 25 (e).

***Supplementary Figure 5. Long radial glia-like fibers in the adult NAc decrease their number with age.***

**a-d**. Confocal images from the avV-SVZ including the NAc of P40, P70, P120, and P180 wild-type mice. Note that fewer Vimentin+/GFAP+ glial fibers can be detected in the NAc at P120 and P180 than at P40 and P70. Yellow arrowheads indicate basal glial processes contacting blood vessels in the NAc. **e**. On the left schematic, illustration of LGE-generated neuronal precursors migrating following RG processes to reach the striatum during embryonic development. On the right panel, adult V-SVZ-derived neuroblasts disperse radially into the NAc following RG-like processes. Abbreviations: ac – anterior commissure; CC – corpus callosum; dSt – dorsal striatum; LGE – lateral ganglionic eminence; lv – lateral ventricle; NAc – nucleus accumbens; Sp – Septum; St – striatum; V-SVZ - subventricular zone. Scale bars in µm: 100 (a-d).

***Supplementary Figure 6. Neuroblasts associate with long radial glia-like fibers in the adult NAc.***

**a**. Coronal brain section of a P65 wild-type mouse. DCX+/PSA-NCAM+ neuroblasts in the NAc in close association with GFAP+ fibers. **a1**. High-magnification (63x) maximum-projection image from the area indicated with the dashed yellow rectangle in a. The area between the dashed orange lines indicate a blood vessel. See Supplementary Movie 5 for further details. **a2**. Single-plane high-magnification (63x) images that evidence a blood vessel wrapped by GFAP+ glial processes. **b**. High-magnification image of DCX+/PSA-NCAM+ neuroblasts located within white matter tracts in the NAc. Abbreviations: ac – anterior commissure; bv – blood vessel; NAc – nucleus accumbens. Scale bars in µm: 50 (a), 20 (a1, a2, b).

***Supplementary Figure 7. Newborn neurons in the adult NAc.***

**a**. High-magnification maximum-projection image of a chain of DCX+ neuroblasts in the NAc of a P70 wild-type mouse, showing expression of the proliferation marker Ki67. Mice received 2 i.p. BrdU injections per day for 5 consecutive days and brains were analyzed 2 days after the last BrdU injection. **b1**, **b2**. Orthogonal views of the same DCX+/BrdU+ neuroblasts shown in a, at 2 levels in the z-axis. **c**. Coronal NAc-containing section of a P98 wild-type mouse injected with BrdU 6 weeks before the staining. **c1**. High-magnification orthogonal views of the yellow dashed rectangle in c. A NeuN+/BrdU+ neuron is shown. **d**. Quantification of the relative frequency of NeuN+/BrdU+ neurons with respect to the distance from the V-SVZ in 14-week old wild-type mice (n = 74 cells from 6 mice). Simple linear regression (R^2^ = 0.5398; slope = -0.0065). Abbreviations: ac – anterior commissure; lv – lateral ventricle; NAcC – nucleus accumbens core; NAcSh – nucleus accumbens shell. Scale bars in µm: 100 (c); 5 (a-b2, c1).

***Supplementary Figure 8. Calbindin staining allows the identification of NAc core and shell boundaries.***

**a.** Schematic of a coronal brain section depicting the NAc layers. Red dashed rectangle denotes the area shown in b. Adapted from the *The Mouse Brain Atlas^92^*. **b.** Coronal image of a P70 mouse brain labeled with CB and DCX antibodies to illustrate the boundaries of NAc core and shell. Abbreviations: ac – anterior commissure; Cpu – caudate putamen; ICj – Calleja Islands; NAcC – nucleus accumbens core; NAcSh – nucleus accumbens shell; OT – olfactory tubercle. Scale bars in µm: 200 (b).

***Supplementary Figure 9. Mature adult-born neurons in the NAc.***

**a.** Sagittal section of the forebrain from NestinCre^ERT2^ ROSA-YFP mice (122-days-old) 58 days after the first tamoxifen injection, immunostained for DARPP-32 and YFP. Top right inset: same brain section stained with DAPI. **a1, a2**. High-magnification images of the yellow dashed rectangles indicated in a. **a1’, a2’**. High-magnification orthogonal views of the cell body of the neuron shown in a1 and a2, respectively. **a3**. High-magnification image of the RMS showing that YFP+ neuroblasts are confined to the RMS. **a4, b**. YFP+ labeled radial glia-like cells in the avV-SVZ present a long basal process that targets the NAc. **c.** Overview of the NAc immunostained for DAPI, biocytin, DARPP-32 and YFP. Inset: Low-magnification image of a coronal brain section of a P130 NestinCre^ERT2^ ROSA-YFP mouse immunostained for DAPI. **d**. High-magnification image of the yellow dashed rectangle in c. **d1**-**d4**. Enlargement of the yellow dashed areas indicated in the picture, showing dendritic spines. **d5**. High-magnification orthogonal views of the biocytin-filled cell body, showing YFP and DARPP-32 expression. **e**. Left: Morphological reconstruction of the biocytin-filled YFP-expressing neuron shown in d. Right: Representative firing pattern of the YFP-labeled MSN neuron recorded in the NAc. Abbreviations: ac – anterior commissure; avV-SVZ – anterio-ventral subventricular zone; CPu – caudate putamen; Cx – cortex; lv – lateral ventricle; NAc – nucleus accumbens; OB – olfactory bulb; OT – olfactory tubercle; RMS – rostral migratory stream; Sp – septum. Scale bars in µm: 1000 (a); 100 (a3), 200 (c), 50 (a4, b, e), 20 (a1, a2, d), 5 (d1-d5). See also Supplementary Movie 7.

***Supplementary Figure 10. Inflammatory and neuropathic pain-induced neurogenesis in the NAc in both hemispheres*.**

**a.** Top: Experimental schematic. Bottom: Quantification of NeuN+/BrdU+ cells in the ipsilateral and contralateral NAc with regard to the operation side in sham or SNI-treated mice (one-way ANOVA, followed by Holm-Sidak multiple comparison test, p= 0.62 for ipsilateral vs contralateral in sham mice and p= 0.57 for ipsilateral vs contralateral in SNI mice, n= 4 mice per group). **a1.** Quantification of NeuN+/BrdU+ cells in the OB and NAc in sham and SNI treated mice. For direct comparison, the data from the NAc shown in (**Figure** **5f, left**) are shown again (*t*-test p= 0.98, n= 8 mice per group for OB and Mann-Whitney test *p= 0.038, n= 8 mice per group for NAc). n.s.: not significant. **b.** Top: Experimental schematic. Bottom: Quantification of NeuN+/BrdU+ cells in the ipsilateral and contralateral NAc with regard to the paw injection, in PBS or CFA-injected mice (one-way ANOVA, followed by Holm-Sidak multiple comparison test, p= 0.54 for ipsilateral vs contralateral in PBS-injected mice and p= 0.50 for ipsilateral vs contralateral in CFA-injected mice, n= 4 mice per group). **b1.** Quantification of NeuN+/BrdU+ cells in the OB and NAc in PBS or CFA injected mice. For direct comparison, the data from the NAc shown in (**Figure** **5f, right**) are shown again (*t*-test p= 0.59, n= 5-6 mice per group for OB, and *t-*test ****p< 0.0001, n= 10 mice per group for NAc).

***Supplementary Figure 11. V-SVZ-origin of newborn neurons in the NAc following inflammatory pain.***

**a**. Experimental schematic to test the origin of NAc adult born neurons after inflammatory pain. **b, c**. Confocal images of the NAc from NestinCre^ERT2^ ROSA-YFP mice 72 days after the first tamoxifen injection. **b1, c1**. Enlarged pictures of the dashed yellow rectangles in (**b, c**) for each channel: DAPI (blue), YFP (green), NeuN (grey), DARPP-32 (red). Abbreviations: ac – anterior commissure; NAc – nucleus accumbens. Scale bars in µm: 100 (b, c).

***Video Captions***

***Supplementary Movie 1.*** Time-lapse video of a P42 5HT3A-EGFP mouse. The area imaged corresponds to Figure 1e and includes the avV-SVZ and the NAc. Lines illustrate the trajectory of recorded cells and dots indicate the end of the trajectory. Scale bar: 50 µm.

***Supplementary Movie 2.*** Rotating video of a coronal section of P65 wild-type mouse. Images were acquired with a 63x objective. The rotating images show the close presence of DCX+ neuroblasts and GFAP+ fibers. The video corresponds to Figure 2a2. Scale bar: 20 µm.

***Supplementary Movie 3.*** Rotating video of a coronal section of P65 wild-type mouse. Images were acquired with a 63x objective. The rotating images show the close presence of DCX+ neuroblasts and GFAP+ fibers. The video corresponds to Supplementary Figure 6a1. Scale bar: 50 µm.

***Supplementary Movie 4.*** 3D stack reconstruction of a coronal section from a P65 wild-type mouse. GFAP+ fibers (green) are in tight association with DCX+ neuroblasts in the NAc. Video related to Figure 2a2. Scale bar: 10 µm.

***Supplementary Movie 5.*** 3D stack reconstruction of a coronal section of P65 wild-type mouse. GFAP+ fibers (green) are in tight association with DCX+ neuroblasts migrating from the V-SVZ to the NAc. Note that GFAP+ processes enwrapped a blood vessel. Video related to Supplementary Figure 6a1, a2. Scale bar: 10 µm.

***Supplementary Movie 6.*** Rotating video of a coronal section of P125 NestinCre^ERT2^ Rosa-YFP reporter mouse. Nine-week-old mouse was injected with tamoxifen. Images were acquired with a 63x objective. The rotating images shows the morphology and dendritic spines of a patched medium spiny neuron injected with biocytin. This video is related to Figure 4h. Scale bar: 50 µm.

***Supplementary Movie 7.*** Rotating video of a coronal section of P125 NestinCre^ERT2^ Rosa-YFP reporter mouse. Nine-week-old mouse was injected with tamoxifen. Images were acquired with a 63x objective. The rotating images shows the morphology and dendritic spines of a patched medium spiny neuron injected with biocytin. This video is related to Supplementary Figure 9d. Scale bar: 20 µm.
