# Supplementary figures and images for: Neurogenesis of medium spiny neurons in the nucleus accumbens continues into adulthood and is enhanced by pathological pain
Source: Mol Psychiatry. 2020 Jul 1;26(9):4616–32. doi: 10.1038/s41380-020-0823-4 (PMC8589654; doi:10.1038/s41380-020-0823-4)

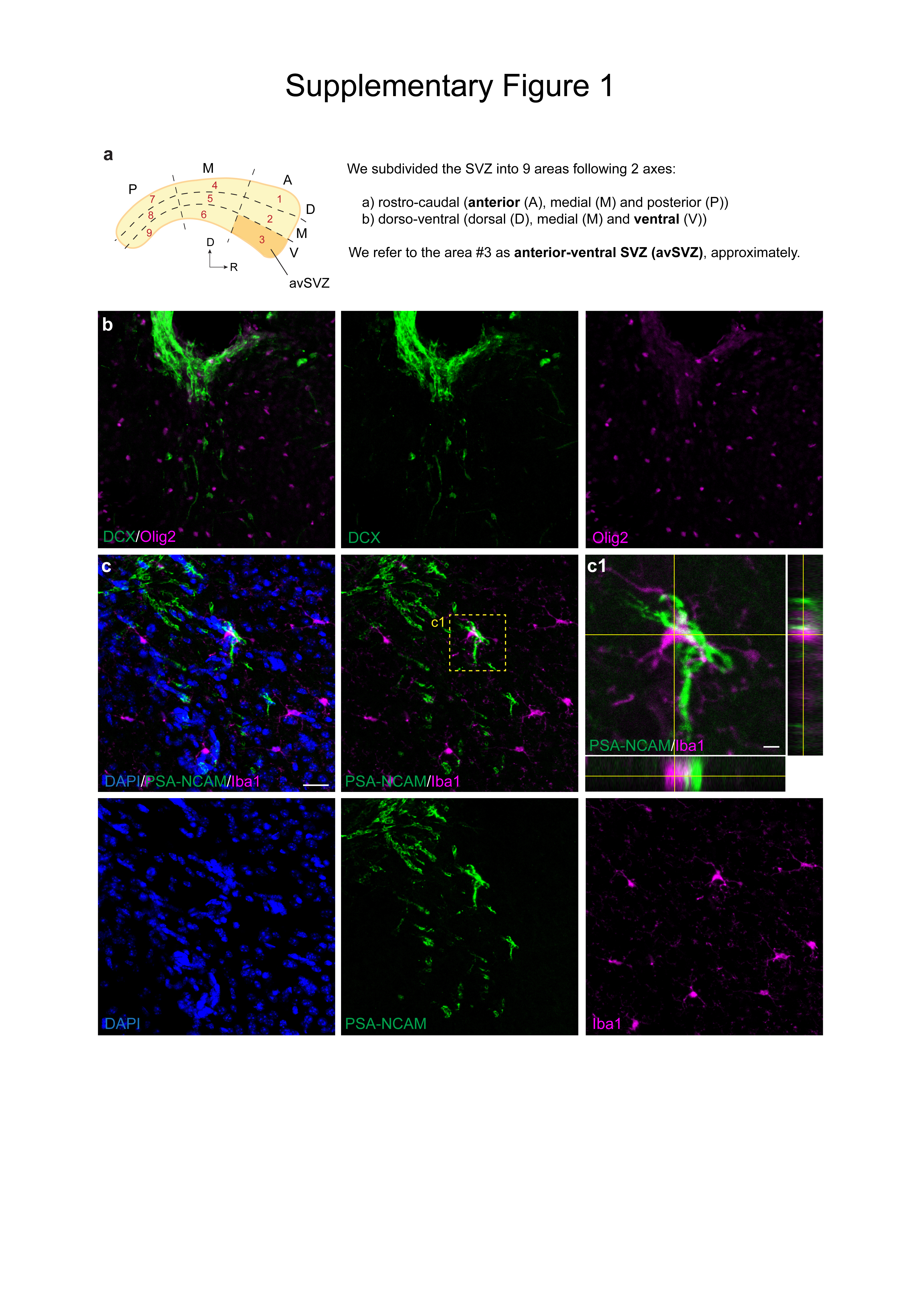

Supplement: Supplementary file 2 — Suppl Fig 1 [file 41380_2020_823_MOESM2_ESM.jpg]

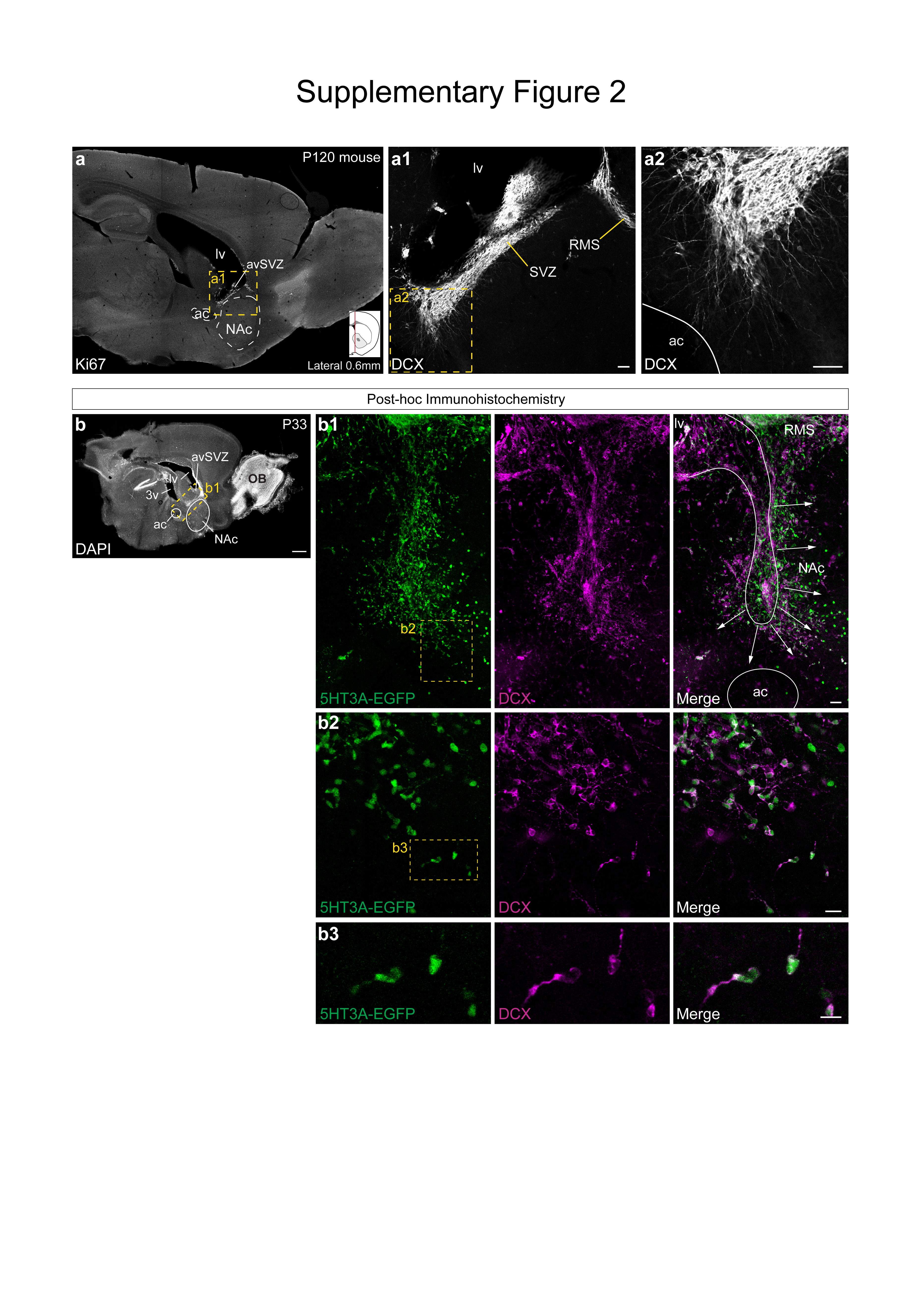

Supplement: Supplementary file 3 — Suppl Fig 2 [file 41380_2020_823_MOESM3_ESM.jpg]

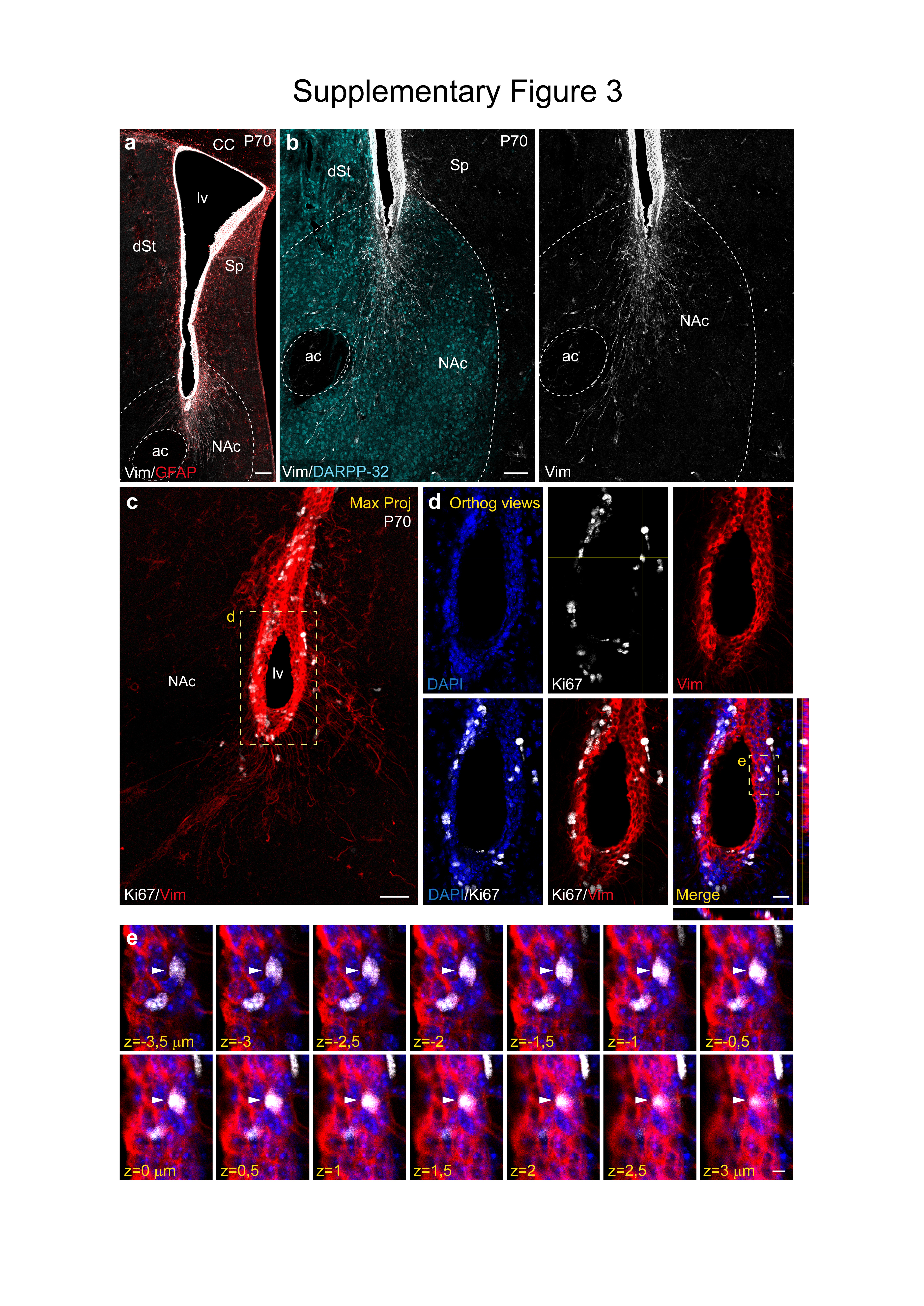

Supplement: Supplementary file 4 — Suppl Fig 3 [file 41380_2020_823_MOESM4_ESM.jpg]

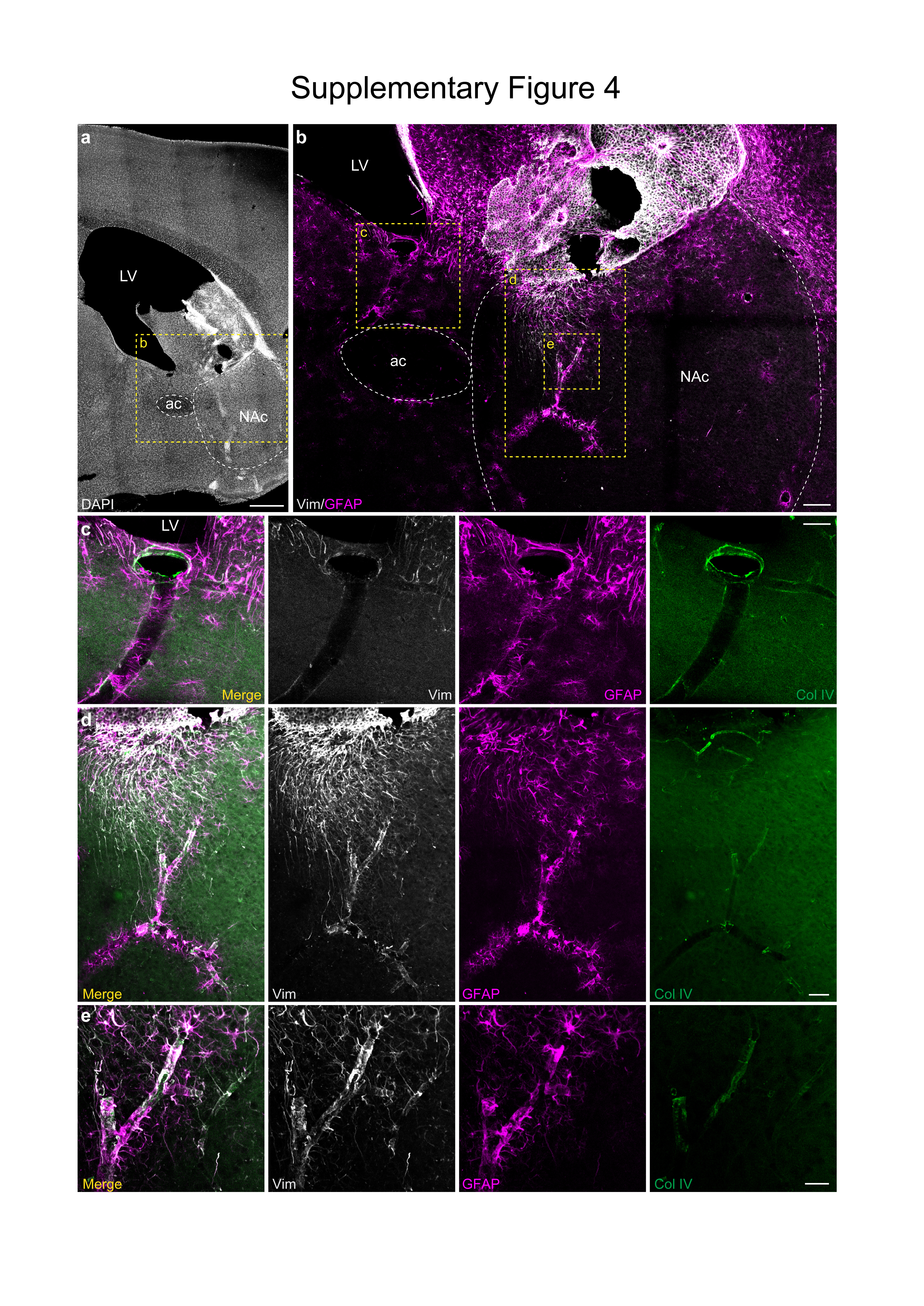

Supplement: Supplementary file 5 — Suppl Fig 4 [file 41380_2020_823_MOESM5_ESM.jpg]

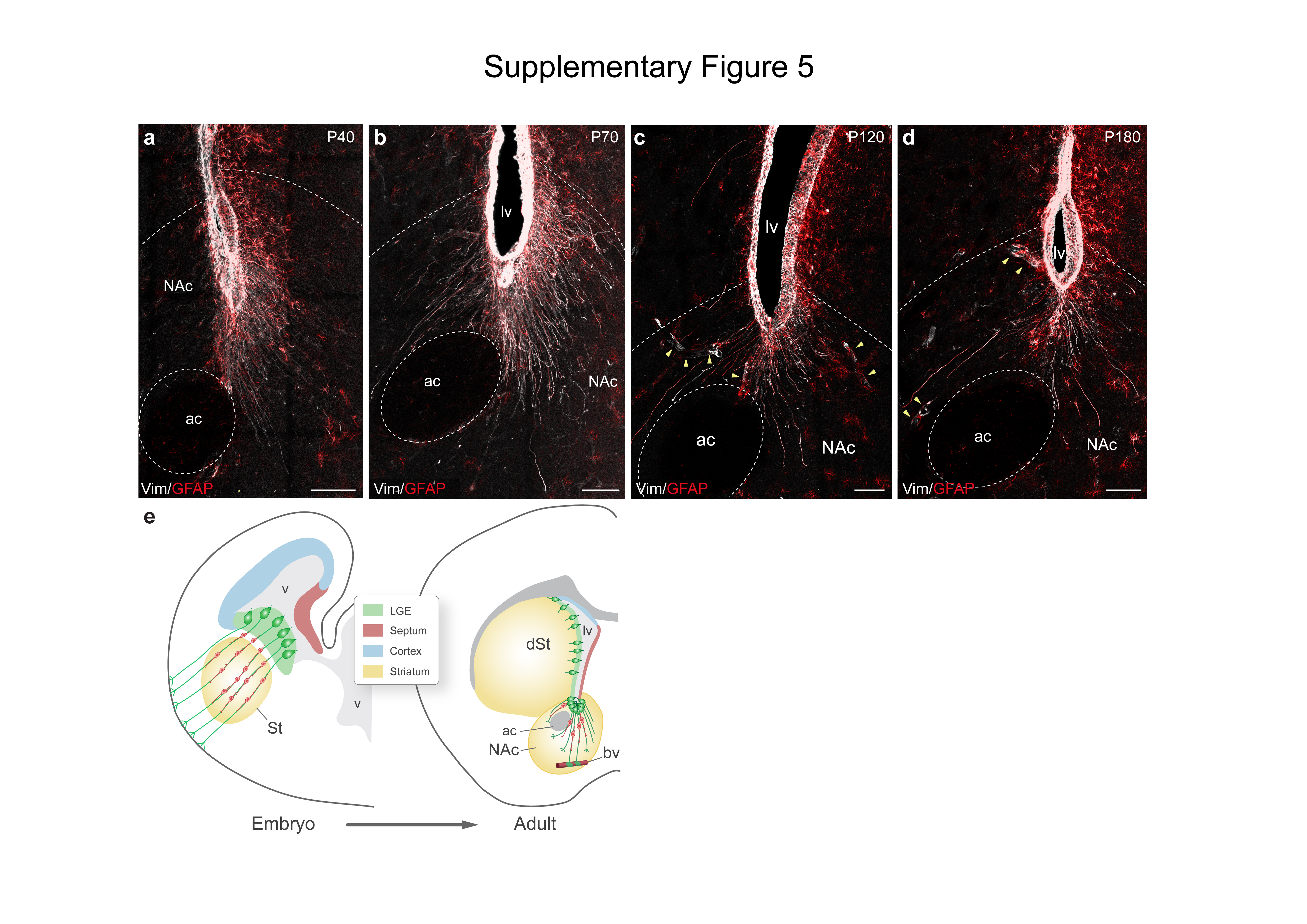

Supplement: Supplementary file 6 — Suppl Fig 5 [file 41380_2020_823_MOESM6_ESM.jpg]

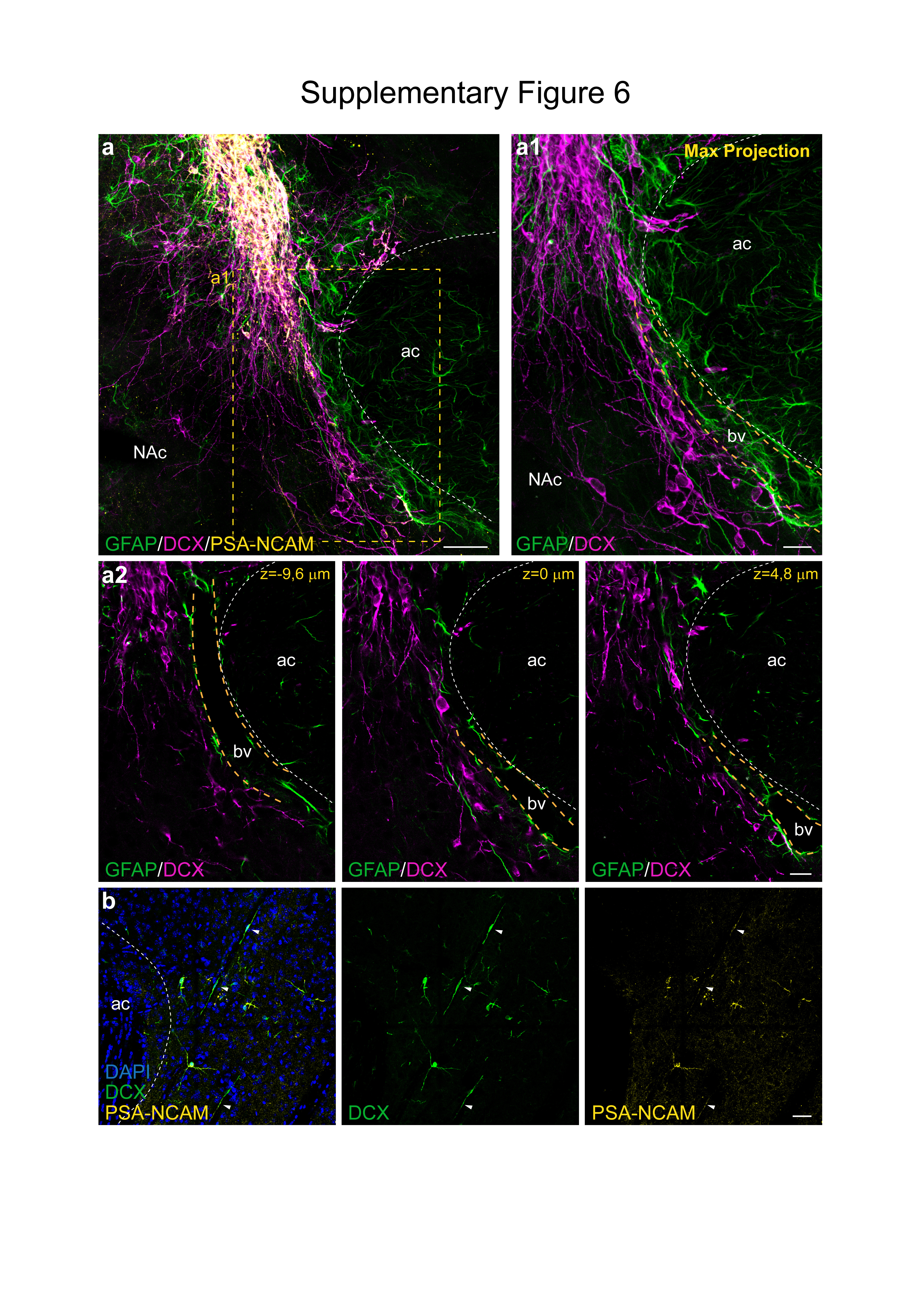

Supplement: Supplementary file 7 — Suppl Fig 6 [file 41380_2020_823_MOESM7_ESM.jpg]

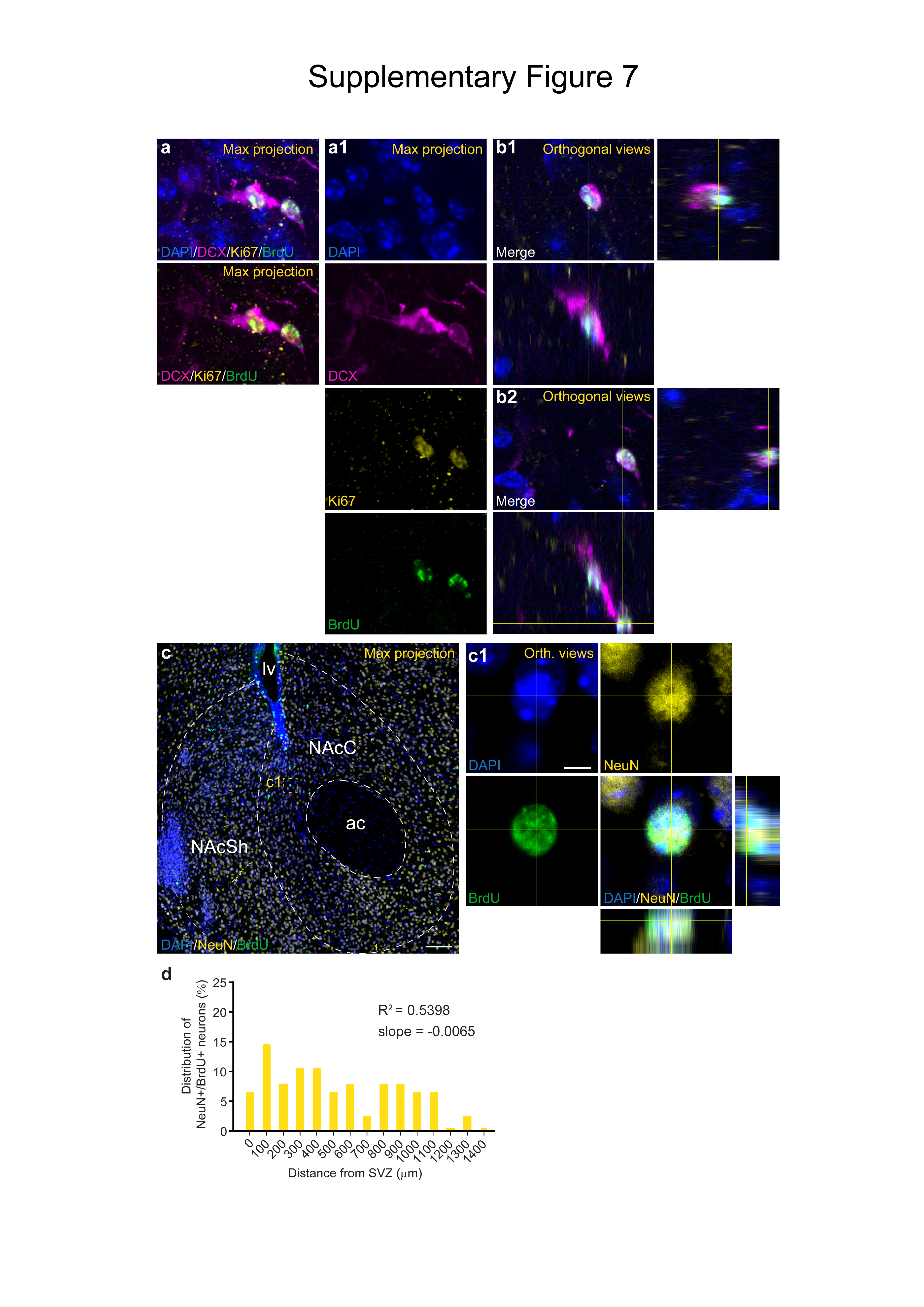

Supplement: Supplementary file 8 — Suppl Fig 7 [file 41380_2020_823_MOESM8_ESM.jpg]

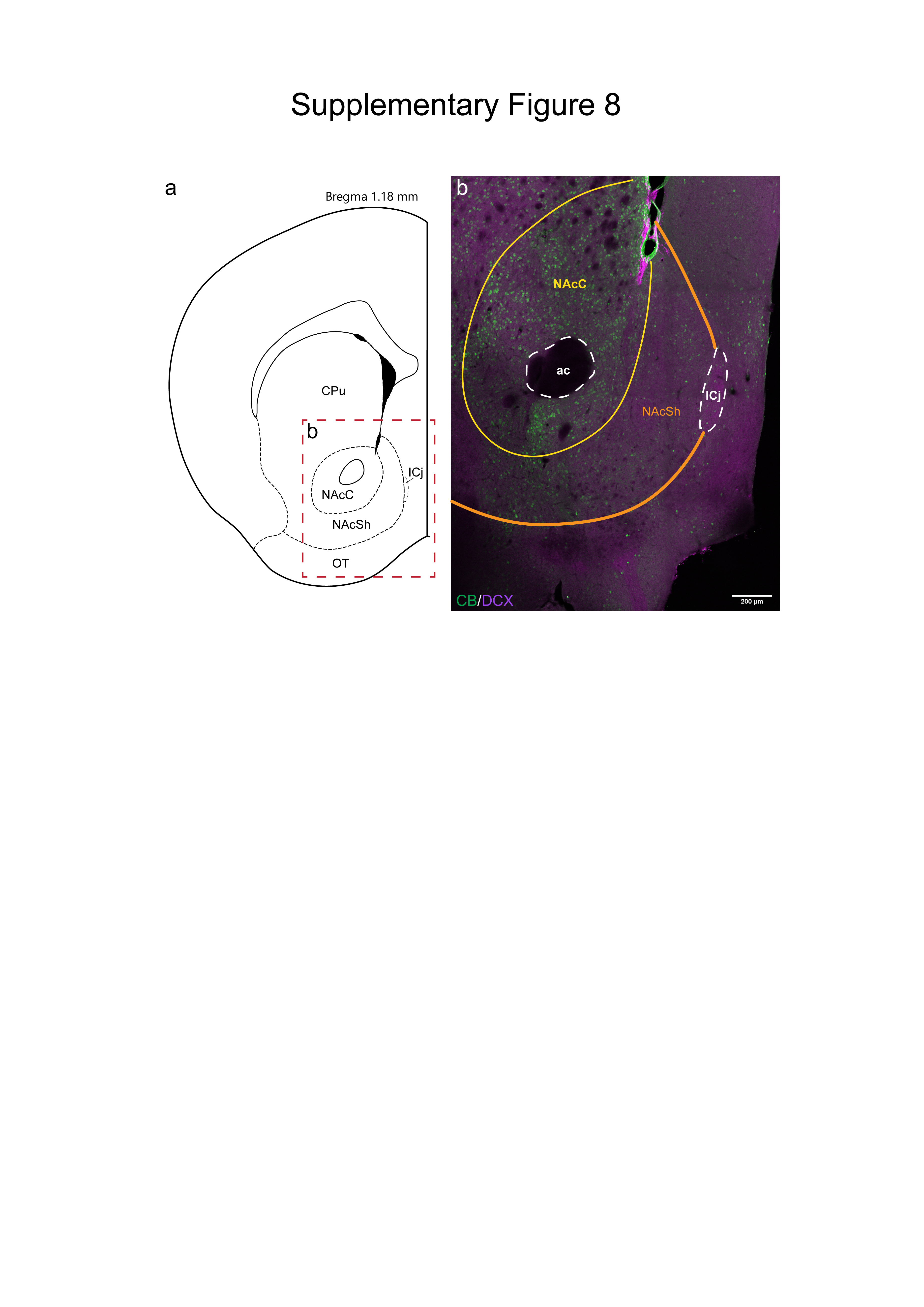

Supplement: Supplementary file 9 — Suppl Fig 8 [file 41380_2020_823_MOESM9_ESM.jpg]

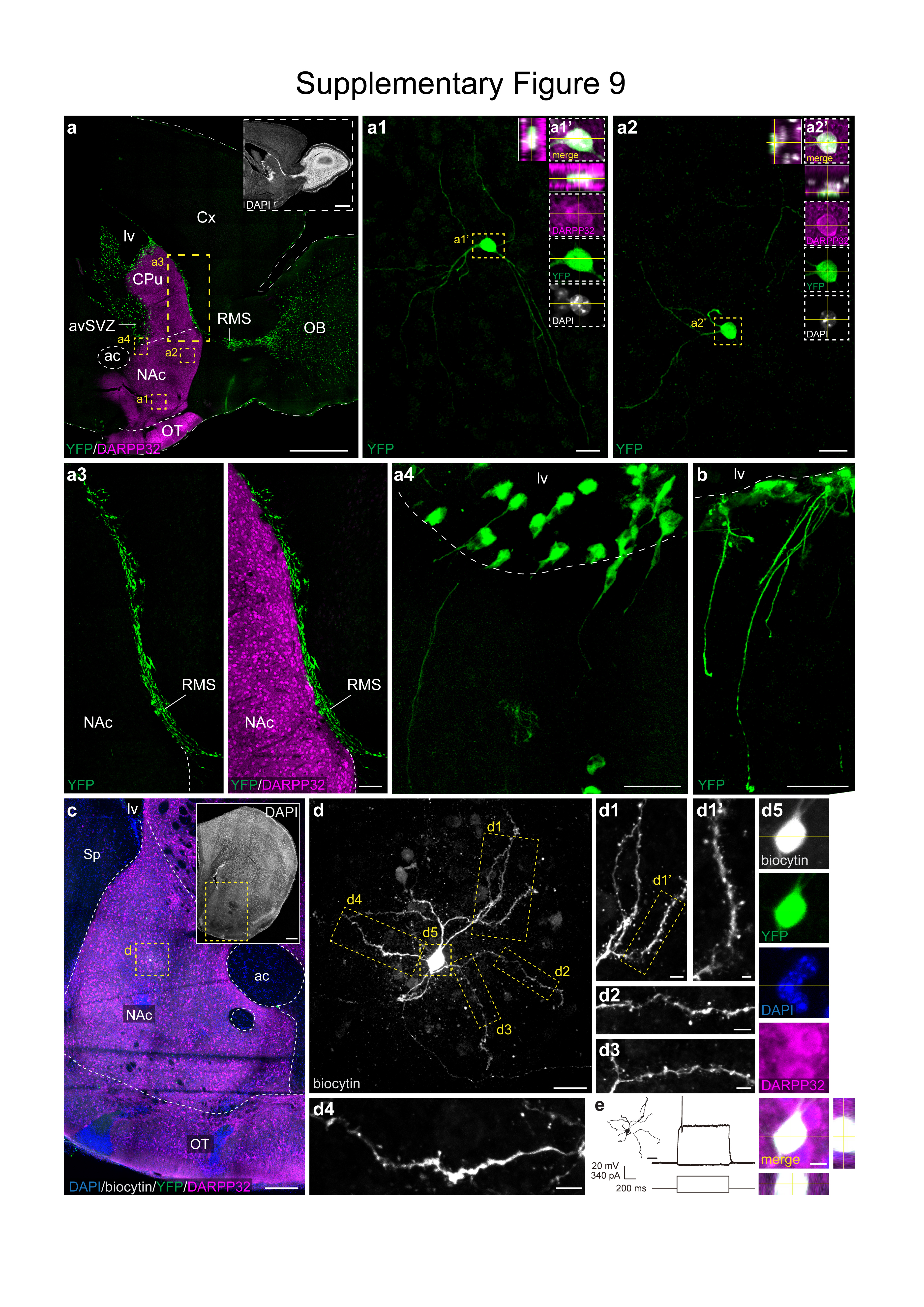

Supplement: Supplementary file 10 — Suppl Fig 9 [file 41380_2020_823_MOESM10_ESM.jpg]

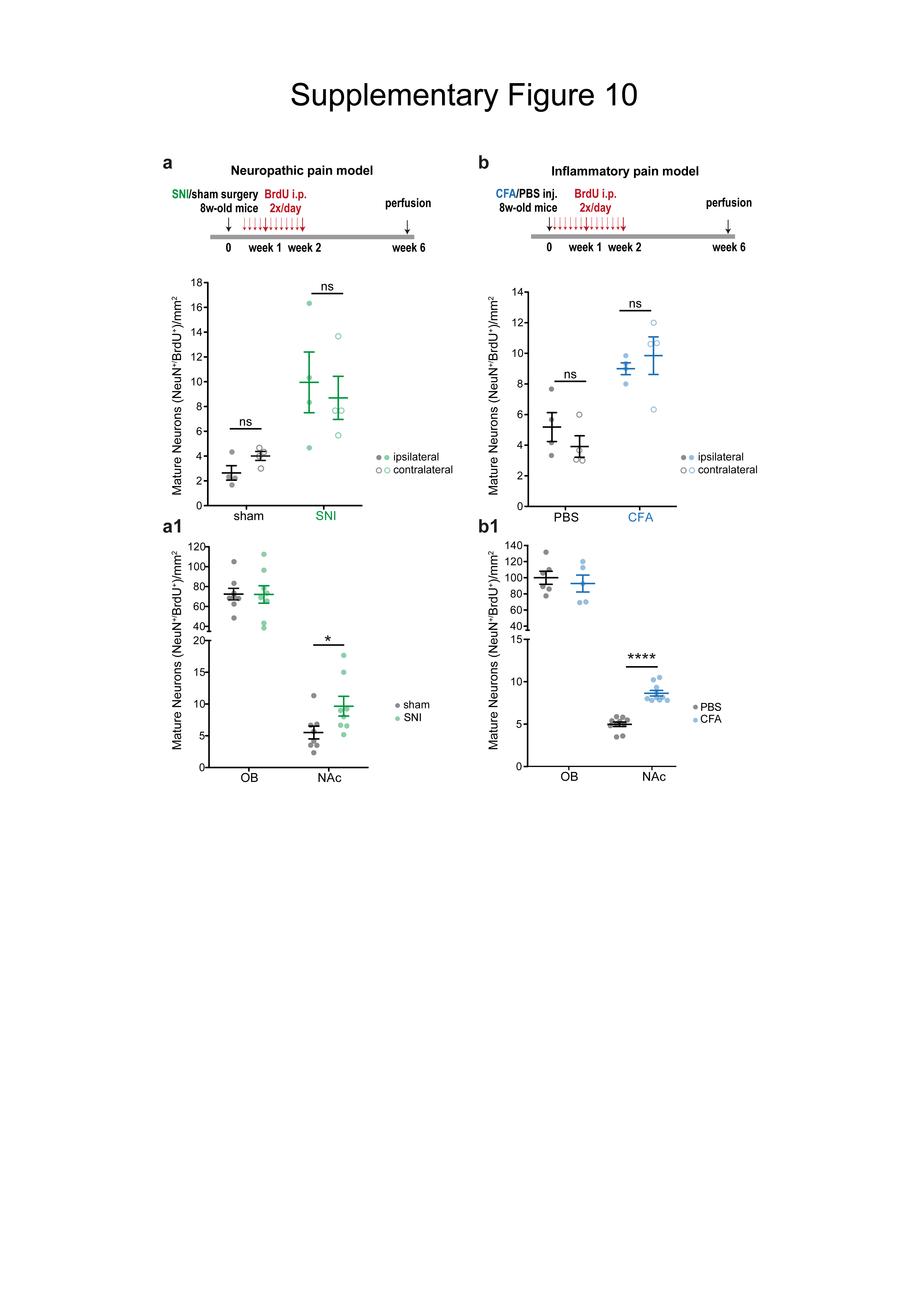

Supplement: Supplementary file 11 — Suppl Fig 10 [file 41380_2020_823_MOESM11_ESM.jpg]

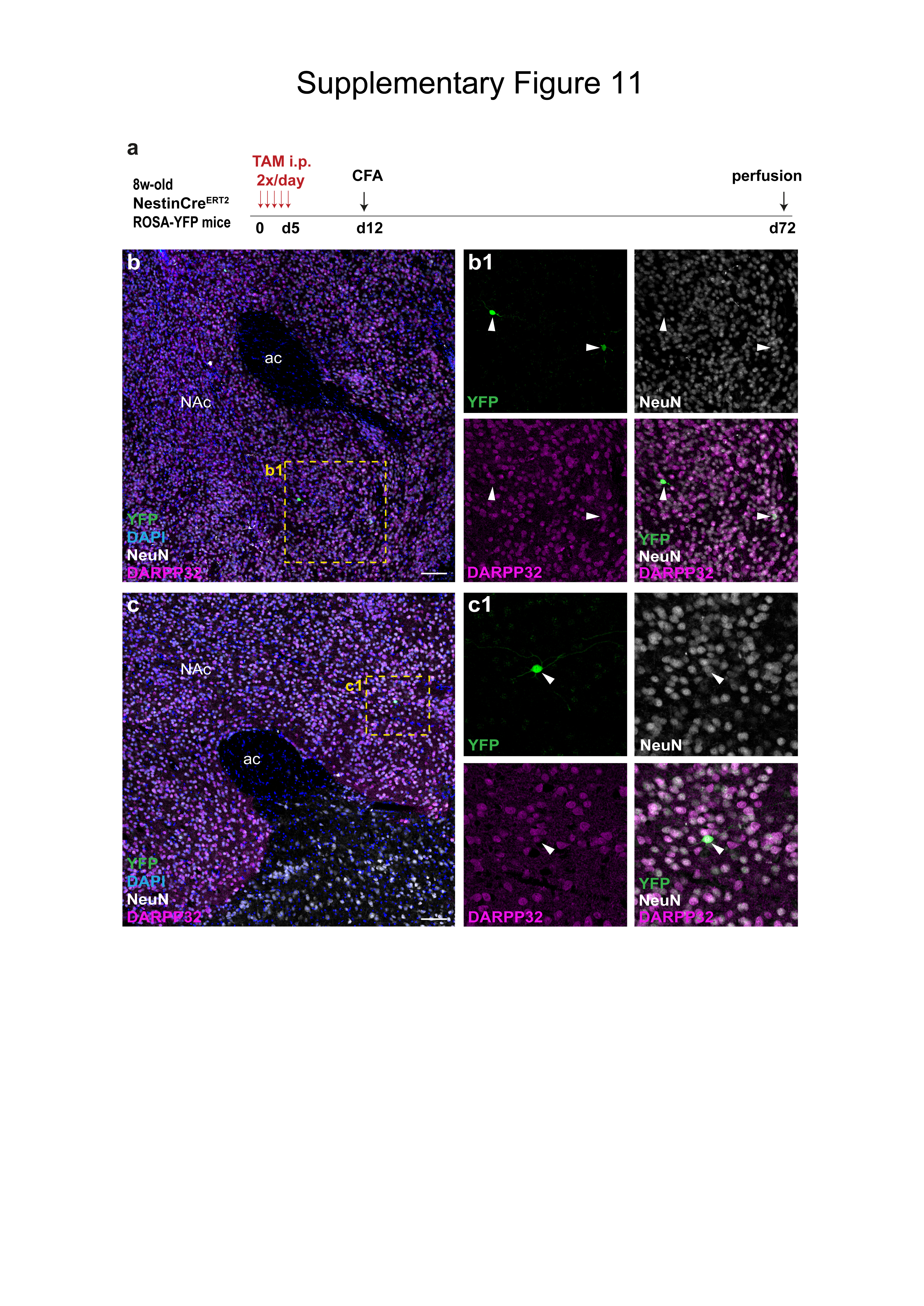

Supplement: Supplementary file 12 — Suppl Fig 11 [file 41380_2020_823_MOESM12_ESM.jpg]
